# Two Distinct Modes of DNA Binding by an MCM Helicase Enable DNA Translocation

**DOI:** 10.3390/ijms232314678

**Published:** 2022-11-24

**Authors:** Martin Meagher, Alexander Myasnikov, Eric J. Enemark

**Affiliations:** 1 Department of Structural Biology, St. Jude Children’s Research Hospital, 262 Danny Thomas Place, Memphis, TN 38105, USA; 2 Department of Biochemistry and Molecular Biology, University of Arkansas for Medical Sciences, 4301 W. Markham St., Little Rock, AR 72205, USA

**Keywords:** ATPase, helicase, DNA replication

## Abstract

A six-subunit ATPase ring forms the central hub of the replication forks in all domains of life. This ring performs a helicase function to separate the two complementary DNA strands to be replicated and drives the replication machinery along the DNA. Disruption of this helicase/ATPase ring is associated with genetic instability and diseases such as cancer. The helicase/ATPase rings of eukaryotes and archaea consist of six minichromosome maintenance (MCM) proteins. Prior structural studies have shown that MCM rings bind one encircled strand of DNA in a spiral staircase, suggesting that the ring pulls this strand of DNA through its central pore in a hand-over-hand mechanism where the subunit at the bottom of the staircase dissociates from DNA and re-binds DNA one step above the staircase. With high-resolution cryo-EM, we show that the MCM ring of the archaeal organism *Saccharolobus solfataricus* binds an encircled DNA strand in two different modes with different numbers of subunits engaged to DNA, illustrating a plausible mechanism for the alternating steps of DNA dissociation and re-association that occur during DNA translocation.

## 1. Introduction

DNA replication is a fundamental life process of duplicating genetic material where the two complementary strands of the DNA double-helix are separated so that each can serve as a template in the synthesis of new DNA [1]. In eukaryotes, archaea, bacteria, mitochondria, and several double-stranded DNA viruses, a ring-shaped hexameric helicase [2,3] enzyme separates the two strands to form a Y-shaped “replication fork” architecture, and it drives this replication fork structure along the DNA. These specialized helicases are termed replicative helicases. The replicative helicases use a strand-exclusion mechanism to separate DNA strands where the ring encircles one strand while excluding the other [4,5,6,7,8]. The helicase ring propagates DNA strand separation as it moves unidirectionally along the DNA with an activity termed translocation [9].

Hexameric ring helicases [2,3] comprise four of six superfamilies (SF) of helicases, as defined by amino acid sequence [9]. Two of the ring helicase superfamilies belong to the AAA+ family of ATPases [10] and translocate the encircled DNA strand with a 3′ to 5′ polarity [11]. These two superfamilies include the SF6 helicases [9], which contain the MCM complexes of eukaryotes and archaea, and the SF3 helicases [12] of some viruses that infect eukaryotic cells, including SV40 large T-antigen [13] and papillomavirus E1 [14]. The other two ring helicase superfamilies belong to the RecA family of ATPases and translocate encircled nucleic acid with a 5′ to 3′ polarity. One is the SF4 helicases, which include the bacterial DnaB helicase [15], the mitochondrial twinkle helicase [16], and the helicases of several bacteriophages such as T7gp4 [17]. The SF5 helicases consist of the bacterial termination factor rho [18], a hexameric helicase that is not a replicative helicase. The polarity differences among these helicase superfamilies dictate fundamentally different replication fork architectures such that the central helicase encircles the leading strand DNA template in eukaryotes, archaea, and some eukaryotic viruses; and it encircles the lagging strand DNA template in bacteria, bacteriophages, and mitochondria.

The eukaryotic MCM ring is unique among known hexameric helicases in that the six constituent subunits all differ from one another, forming a heterohexameric Mcm2-7 complex [19]. The subunits of this complex occupy a defined arrangement around the ring [20,21]. The MCM rings of archaeal organisms, such as *Saccharolobus solfataricus*, form a homohexameric ring. As discussed by Rzechorzek et al., the specialized subunits of eukaryotic Mcm2-7 may not have evolved to perform DNA translocation and DNA strand separation activities because all other hexameric helicases can fulfill these functions as homohexamers [22]. Instead, the specialization of the six eukaryotic Mcm2-7 subunits may facilitate functions other than translocation, such loading, activation, and termination [22]. Indeed, we will illustrate that the core ATPase and DNA-binding structure of all MCM proteins are extremely similar, suggesting that all can act similarly, as in a homohexamer, in the fundamental ATPase and DNA-binding activities of DNA translocation.

The MCM rings of eukaryotes and archaea interact with additional factors [23,24] to form a larger complex, known as the CMG complex [23] in eukaryotes. The additional components of this complex, Cdc45 and GINS [23], are essential genes and essential for in-vitro origin-dependent DNA replication [25]. These factors are not required to translocate DNA or to unwind DNA because the six Mcm2-7 proteins alone are sufficient to unwind DNA biochemically [26]. Similarly, archaeal MCM proteins alone are sufficient to unwind DNA biochemically [11]. Collectively, these indicate that the six-member MCM ring is a minimal complex for DNA translocation/unwinding and that the added factors serve specialized, essential functions that allow cells to achieve the intricate regulation needed for origin-dependent DNA replication.

All hexameric replicative ring helicases share basic mechanistic features and analogy to F1-ATPase [2]. F1-ATPase has three catalytic ATPase sites at subunit interfaces, and the activities of these sites manipulate a stalk within the central channel (and vice versa) [27]. In this “binding site change” mechanism [28], the three catalytic ATPase sites around the ring sequentially pass through tight, loose, and open states in response to ATP-binding, ATP-hydrolysis, and release of ATP-hydrolysis products. These changes at the intersubunit ATPase sites cause the subunits to move with respect to each other and rotate the γ-stalk bound within the central channel. The strictly sequential hydrolysis of ATP around the ring is described as “rotary catalysis” [29,30]. Early EM experiments illustrated that bacteriophage T7gp4 forms a ring-shaped structure [4,31], and this provided a basis for functional analogies between hexameric ring helicases and F1-ATPase with helicases using a binding-site change mechanism [2]. A crystal structure of T7gp4 illustrated different conformations among the ATPase sites that correlated with the heights of the DNA-binding modules positioned in the central channel [32]. Collectively, the structure indicated the basis for T7gp4 to employ a binding site change mechanism at the ATPase sites to manipulate nucleic acid bound within the central channel. A molecular level mechanism that hexameric rings use to pull an encircled strand of DNA through the central channel was illustrated with an X-ray crystal structure of the papillomavirus SF3 helicase E1 bound to encircled single-stranded DNA (ssDNA) and nucleotide co-factors at the subunit interfaces [5]. The structure showed that each E1 subunit binds one nucleotide of ssDNA in a spiral staircase.

The crystal structure of papillomavirus E1 bound to ssDNA and nucleotide cofactors illustrates that the six pre-sensor-1 β-hairpins (PS1β) of the AAA+ architecture collectively form a spiral staircase with the individual hairpins binding one nucleotide of ssDNA. The position of each hairpin in the staircase correlates with the conformation of the ATPase site of the subunit with hairpins at the top of the staircase correlated with an ATP configuration, the hairpins in the middle correlated with ADP, and the hairpins at the bottom associated with an empty ATPase state. In a straightforward mechanism for DNA translocation, each hairpin of the staircase maintains continuous association with one nucleotide of DNA, and the overall staircase descends upon ATP hydrolysis to pull the strand downward through the ring pore. Next, the hairpin at the bottom of the staircase releases its associated nucleotide of DNA and moves to the top of the staircase upon binding a new ATP molecule to bind the next incoming nucleotide of DNA. Thus, each hairpin progresses stepwise from the top of the staircase to the bottom to escort one associated nucleotide of ssDNA through the central channel. The ATP hydrolysis cycle proceeds sequentially around the ring in a sequential rotary mechanism analogous to that of F1-ATPase [33]. In one complete cycle around the hexamer ring, the six hairpins collectively move six nucleotides of ssDNA through the central channel. The mechanism can be described as “hand-over-hand”, similar to six hands pulling on a rope followed by movement of the bottom hand to the top.

An analogous spiral staircase has been observed for all other superfamilies of hexameric ring helicase. The superfamilies vary in the number of nucleotides of DNA bound per subunit within the staircase. SF4 and SF6 helicases bind encircled ssDNA in a two-nucleotide-per-subunit staircase [7,34]. The SF5 helicase rho binds RNA in a one-nucleotide-per-subunit increment [35]. The overall translocation mechanisms are highly analogous with the collective staircase descending followed by the bottom subunit hopping to the top of the staircase. The different increments of binding lead to different numbers of nucleotides translocated in a full cycle. One full cycle translocates 6 nucleotides for SF3 [5] and SF5 [35] helicases and translocates 12 nucleotides for SF4 [7] and SF6 [36] helicases.

The fundamental staircase mechanism has two oscillating stages. First, the collective staircase descends, associated with ATP hydrolysis. Second, the subunit at the bottom step of the staircase disengages from DNA, moves ahead of the other subunits and re-engages binding to DNA at the top of the staircase. These discrete steps involve different numbers of subunits engaged in DNA-binding. The two distinct hexamers in the papillomavirus E1–DNA crystal structure [5] illustrated such DNA-engagement differences with 6 subunits of one hexamer binding DNA and 5 subunits of the other hexamer binding DNA. Our earlier crystal structure of an archaeal MCM hexamer bound to ssDNA [36] had only one hexamer in the asymmetric unit and, thus, showed only one mode of DNA engagement. Using cryo-EM, we now find that this MCM complex has two modes of DNA-engagement: one with 11-nucleotides of ssDNA and the other with 9-nucleotides of ssDNA. Together, these illustrate the oscillating DNA-binding and DNA-release events that enable DNA translocation by this SF6 family of helicase that operates at the replication forks of eukaryotes and archaea.

## 2. Results

To examine the mechanism of DNA translocation by the MCM hexameric helicase, we determined cryo-EM structures of a protein construct of the MCM protein from the archaeal organism *Saccharolobus solfataricus* (*Sso*MCM) in complex with the ATP analog ADP-BeF_3_, magnesium, and oligonucleotide. In attempting to reveal different aspects of DNA manipulation by the MCM helicase, cryo-EM structures were determined with three different oligonucleotides of different lengths and potential for secondary structure. Despite the differences of the oligonucleotides used for complex formation, each sample showed highly similar behavior with two distinct structural classes. Higher resolution structures of these classes were obtained by merging all particles from the respective classes of the three distinct samples. The individual structures and the high-resolution structures obtained from the merged particles are described below.

### 2.1. Overall Architecture

**Figure 1:**
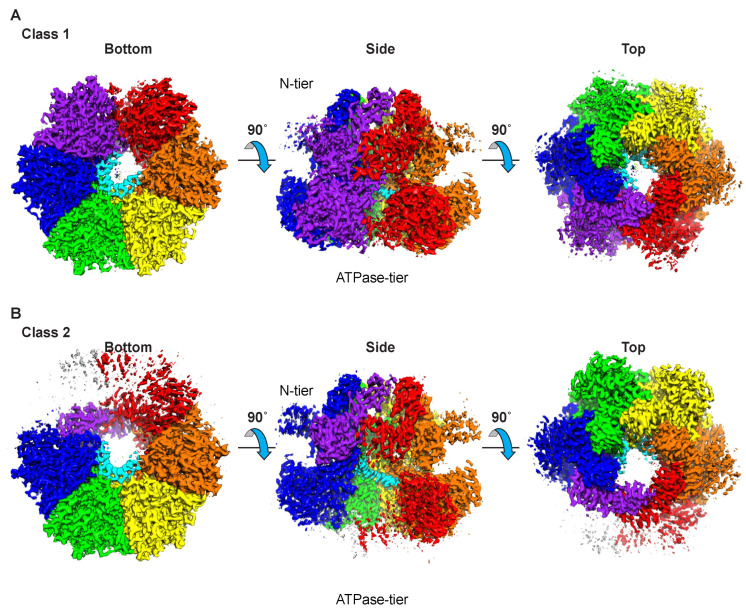
Overall architectures of the two structural classes of MCM–ssDNA–MgADP-BeF_3_. Three orthogonal, consistent views are illustrated for the two classes. The “side” view is perpendicular to the central channel with the N-terminal tier on top and the ATPase tier on the bottom. The “bottom” view is parallel to the central channel from the ATPase tier side. The “top” view is parallel to the central channel from the N-terminal tier side. The illustrated maps were obtained by refinement of the merged particles of the respective classes obtained for three different oligonucleotides (see Figures S2–S4). Both classes consist of a two-tiered hexameric ring that binds single-stranded DNA at the larger ATPase tier. The defined views place the N-terminal tier at the top of the complex and the ATPase tier at the bottom. Each figure panel was prepared with Chimera [47]. (**A**) The final sharpened map following homogeneous refinement of class 1 is colored by subunit proximity to the atomic model coordinate file. Class 1 has 6 well-defined domains at both tiers. The peripheral helical bundle subdomains of the N-terminal tier appear less ordered than the other subdomains. (**B**) The final sharpened map following homogeneous refinement of class 2 is colored by subunit proximity to the atomic model coordinate file. Class 2 has 6 well-defined domains at the center of the N-terminal tier and 5 well-defined ATPase domains. The ATPase domain of the purple subunit is poorly ordered. Two of the peripheral helical bundle domains of the N-terminal tier are poorly ordered. Maps are displayed by Chimera [47] at contour level 1.4 for class 1 and at contour level 1.2 for class 2 to emphasize the well-ordered DNA-bound portion.

**Figure 2:**
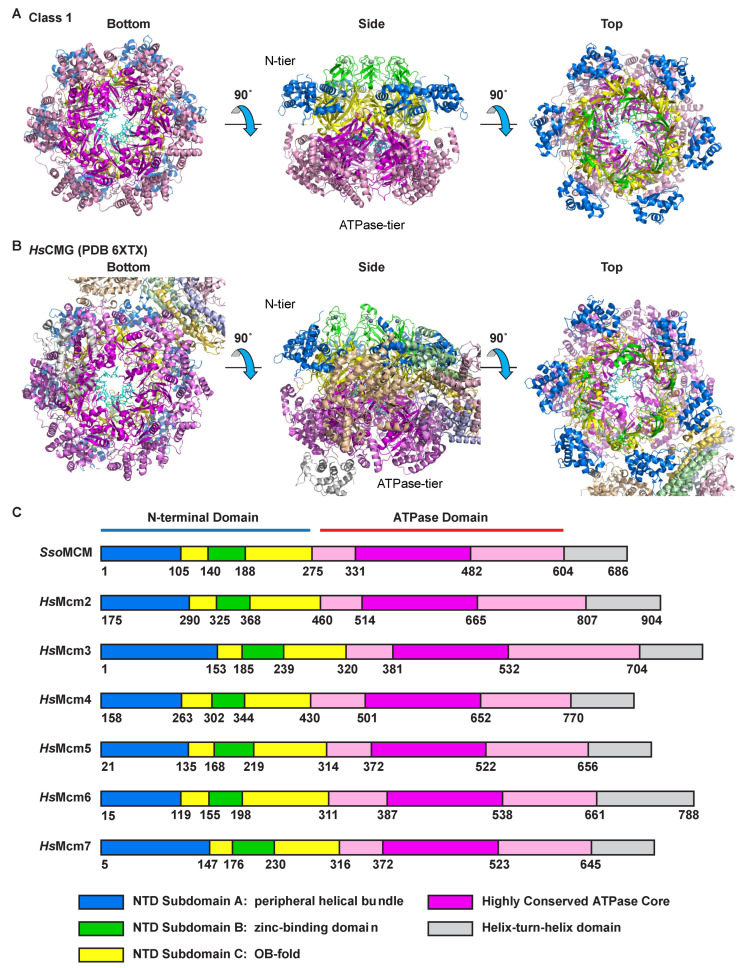
Subdomain architecture of MCM proteins. Subdomains are as assigned previously [44]. (**A**) Three perpendicular views of the subdomain architecture of *Sso*MCM within the MCM–ssDNA–MgADP-BeF_3_ class 1 structure. The “side” view is perpendicular to the central channel with the N-terminal tier on top and the ATPase tier on the bottom. The “bottom” view is parallel to the central channel from the ATPase tier side. The “top” view is parallel to the central channel from the N-terminal tier side. The model obtained from the merged particle set is illustrated. From the N-terminal tier, the peripheral helical bundle subdomain is in blue, the OB-fold subdomain in yellow, and the Zn-binding subdomain in green. The ATPase tier is in pink and magenta with the core contiguous 152-amino-acid segment (see Figure S5) in magenta and other portions in pink. The bound strand of DNA is in cyan stick representation. (**B**) Three perpendicular views of the subdomain architecture of the human CMG–ssDNA structure (PDB 6XTX [22]) emphasizing Mcm2-7. Colors are similar to (**A**) with the peripheral helical bundle subdomain in blue, the OB-fold subdomain in yellow, and the Zn-binding subdomain in green. Subdomains are as assigned previously [44]. The ATPase tier is in pink and magenta with the core contiguous 152-amino-acid segment in magenta. The bound strand of DNA is in cyan stick representation. Cdc45 and the tetrameric GINS complex are at the periphery and colored wheat, light pink, light blue, light green, and yellow–orange. (**C**) Block diagrams indicating the amino acid sequence boundaries of each subdomain for the proteins depicted in (**A**,**B**). (**A**,**B**) were prepared with Pymol [48].

Two consistent and distinct classes of MCM–ssDNA structure are observed for the complexes of three different oligonucleotides (Figure 1 and Figures S1–S4; Table 1). Both classes consist of a two-tier structure analogous to other reports of MCM hexameric ring structures [11,21,37,38,39,40,41,42,43]. The tiers consist of an N-terminal tier with three subdomains defined previously [44] and a C-terminal ATPase tier (Figure 2). The tiers and fundamental subdomain architectures are also adopted by eukaryotic CMG complexes, as illustrated with the human CMG–DNA high-resolution cryo-EM structure (PDB 6XTX; EMD-10619 [22]) (Figure 2B). In the N-terminal tier, the OB-fold β-barrel subdomains (yellow, Figure 2) of the six subunits collectively form an approximately 6-fold symmetric ring at the heart of the N-terminal tier. Helical bundle subdomains attached to the OB-fold sit at the periphery of the N-terminal tier ring (blue, Figure 2). Zinc-binding subdomains attached to the OB-fold ring extend the length of the channel above the OB-fold subdomains (green, Figure 2). In eukaryotes, the zinc-binding domain of Mcm3 is unique because it lacks the residues that bind a zinc ion in all the other subunits (see Figure 3B of [45]) while maintaining a topology equivalent to the subdomains that do bind a zinc ion (See EMD-6338 [21] and PDB 6EYC [46]). Within the N-terminal tier, all interactions between the subunits involve the OB-fold subdomains, suggesting this portion of the N-terminal tier is the most rigidly structured. Although the zinc-binding subdomains are adjacent to each other, they are not close enough to form hydrogen bonds. The zinc-binding subdomains are less ordered than the OB-fold subdomains (Figure 1 and Figures S2–S4), likely due to the absence of such intersubunit interactions. Similarly, the helical bundle subdomains of the N-terminal tier do not interact with neighboring subunits and are more weakly ordered than the OB-fold subdomains. In the case of eukaryotic CMG, the peripheral subdomains of Mcm2 and Mcm5 form all of the MCM–Cdc45 interactions (Figure 2B), and the peripheral subdomains of Mcm5 and Mcm3 form nearly all of the MCM–GINS interactions (Figure 2B).

The C-terminal ATPase tier forms a ring encircling a strand of ssDNA (pink and magenta, Figure 2). The core of the ATPase domain is highly conserved among all archaeal and eukaryotic MCM proteins. With rare and extremely minor exceptions, this core structure is an invariantly structured contiguous stretch of 152 amino acids without insertion or deletion. Of 4 archaeal and 54 eukaryotic MCM sequences, six have a one-residue deletion for a 151-amino acid core AAA+ portion (Figure 2 and Figure S5). This invariantly structured region includes all core β-strands of the AAA+ fold, the Walker-A, Walker-B, and arginine finger ATPase motifs, and the helix-2-insert and pre-sensor-1-β DNA-binding hairpins. Hence, the core portion of all MCM hexameric rings that fulfills ATPase and DNA-binding activities is very symmetric, even though the subunits may differ around the ring. Further details of the key parts of this core AAA+ unit are provided in sections below.

Each ATPase subunit projects two hairpins into the central channel, and these hairpins bind ssDNA in a spiral staircase. Previously, we identified the residues of the MCM hairpins that interact with the sugar–phosphate backbone of ssDNA [36]. The higher resolution of the present structures allows more precise assignment of the specific oxygen atoms of the DNA backbone that interact with these residues. In contrast to the OB-folds of the N-terminal tier, the intersubunit interactions in the ATPase tier are not uniformly identical at each interface around the ring. The different interactions correlate with binding of nucleotide at the bipartite ATPase site formed at the subunit interfaces. The distribution of the different ATPase site configurations is one feature that defines the difference between the classes of structure observed. The most prominent difference between the two classes is that a different number of subunits engage in binding DNA. One class has six subunits engaged in DNA-binding while the other has five subunits engaged. These structural classes are described further below.

### 2.2. Class 1: Six Subunits Bound to ssDNA

**Figure 3:**
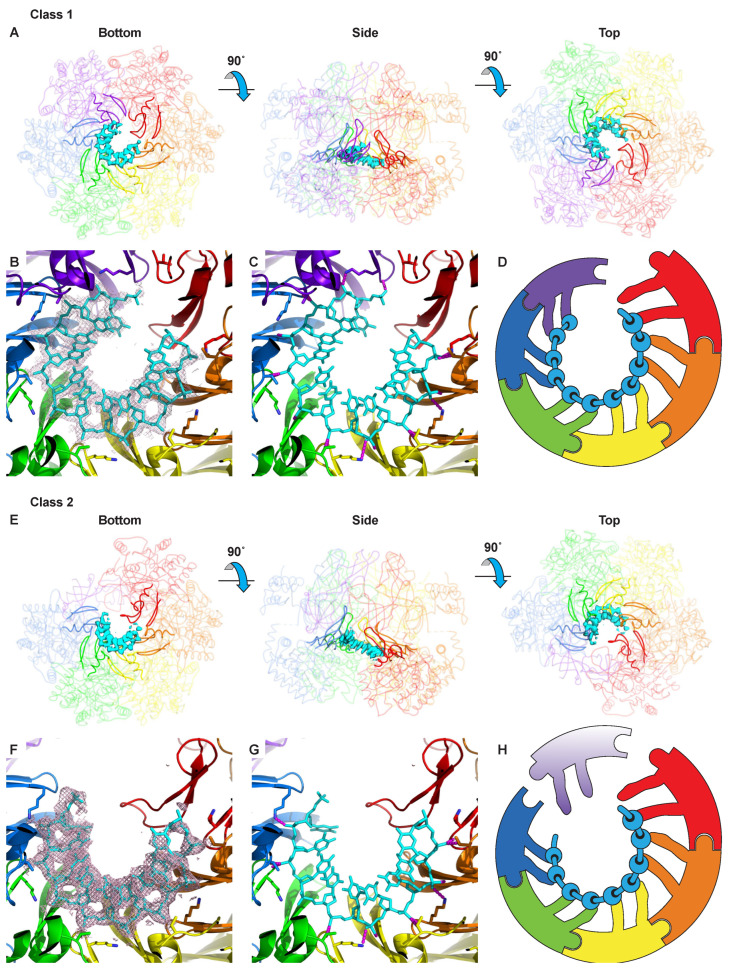
DNA binding in the two structural classes of MCM–ssDNA–MgADP-BeF_3_. The illustrated maps and models were obtained by refinement of the merged particles of the respective classes obtained for three different oligonucleotides (see Figures S6–S8). (**A**) Three orthogonal views of class 1 with the ssDNA portion of the map depicted in cyan and the protein model in ribbon representation. The two DNA-binding hairpins of each subunit are shown in opaque, and the other protein features are partially transparent. The panel view is identical to Figure 1A,B. (**B**) Zoom-in view of the class 1 model illustrating the DNA-binding hairpins and ssDNA. All 4.0-sigma density within 2.5 Å of the modeled DNA shown in mesh. Eleven nucleotides of ssDNA are observed for class 1. (**C**) An identical view to (**B**) illustrating all MCM–DNA hydrogen bonding interactions (<3.5 Å) in dashed magenta with density mesh removed for clarity. (**D**) Cartoon summarizing the sequential interactions of MCM DNA-binding hairpins with the 11 DNA nucleotides, each represented by a bead on a string. (**E**) Three orthogonal views of class 2 with the ssDNA portion of the map depicted in cyan and the protein model in ribbon representation. The two DNA-binding hairpins of each subunit are shown in opaque, and the other protein features are partially transparent. The panel view is identical to Figure 1A,B. (**F**) Zoom-in view of the class 2 model illustrating the DNA-binding hairpins and ssDNA. All 4.0-sigma density within 2.5 Å of the modeled DNA shown in mesh. Nine nucleotides of ssDNA are observed for class 2. (**G**) An identical view to (**B**) illustrating all MCM–DNA hydrogen bonding interactions (<3.5 Å) in dashed magenta with density mesh removed for clarity. (**H**) Cartoon summarizing the sequential interactions of MCM DNA-binding hairpins with the 9 DNA nucleotides, each represented by a bead on a string. The poorly ordered purple ATPase domain is represented with gradient shading. (**A**,**E**) were prepared with Chimera [47], and (**B**,**C**,**F**,**G**) were prepared with Pymol [48].

The first class of structure is well-structured throughout and binds a very well-ordered segment of 11 nucleotides of ssDNA within the central channel at the ATPase tier (Figure 3A–D, Figure S6A–D, Figure S7A–D and Figure S8A–D). The N-terminal tier is approximately 6-fold symmetric. The ATPase tier is not 6-fold symmetric because the subunits form a helical “staircase” that binds encircled ssDNA. This structural class is similar to our previous DNA-bound crystal structure [36] with the most notable difference being that the central pore is slightly more constricted in the crystal structure (Figure S9). The helical structure formed by the ATPase domains necessitates a discontinuity between the top of the staircase and the bottom. This fundamental mode of binding where one strand of nucleic acid is bound by a staircase of ATPase domains has thus far been universally adopted in all high-resolution structures of hexameric helicases encircling nucleic acid [5,7,22,34,35,36]. In this specific class of structure, the staircase of ATPase domains consists of five subunits that bind DNA equivalently via two hairpins (see below). The sixth ATPase domain (red, Figure 3) interacts with ssDNA at the at the bottom of the staircase with just one hairpin. This subunit also loosely interacts with DNA at the top of the staircase. This positioning of the sixth subunit intermediate between the top and bottom of the staircase causes the overall ATPase tier to appear more closed than if the helical arrangement were not disrupted. However, a larger gap between one pair of subunits is clearly present, which correlates with a differently configured ATPase site, described in a section below.

The MCM ATPase domain projects two hairpins into the central channel to bind DNA. These two modules are known as the “pre-sensor-1-β” hairpin (ps1β) and the “helix-2-insert” hairpin (h2i) based on their position in the AAA+ sequences [10]. Four residues, two from each hairpin, bind three consecutive phosphates of ssDNA, as observed previously [36] (see Figure 3C,D). For the ps1β, the ammonium group of K430 forms a salt-bridge with a DNA phosphate, and the main-chain amide of A431 interacts with the phosphate of the neighboring DNA nucleotide. Notably, this mode of binding is identical to that formed by the ps1β of papillomavirus E1 helicase and DNA [5] (see Supplementary Figure S2 of [36]). Similarly, the main-chain amide of h2i V377 interacts with one DNA phosphate while the sidechain of h2i T369 interacts with the phosphate of the neighboring residue.

### 2.3. Class 2: Five Subunits Bound to ssDNA

The second class of structure also forms a spiral staircase of hairpins that bind a well-ordered nine-nucleotide segment of ssDNA within the central channel (Figure 3E–H, Figure S6E–H, Figure S7E–H and Figure S8E–H). The ATPase tier differs significantly from class 1 and the prior crystal structure [36] in that only five subunits participate in DNA binding. Four well-ordered ATPase domains (blue, green, yellow, orange in Figure 3E–H) coordinate DNA in a mode indistinguishable from the DNA-binding of class 1. Similar to class 1, the subunit at the bottom of the staircase (red, Figure 3E–H) binds DNA with just one of its DNA-binding hairpins. This ATPase domain is much more poorly ordered, likely because it is not tethered to other parts of the structure as extensively as the first four ATPase domains. The sixth ATPase domain is quite disordered and is likely not tethered to the DNA or any of the neighboring subunits. The flexibility of ATPase domains not involved in binding DNA or ATP may be functionally important, discussed further in the discussion section. The subunits that participate in binding DNA use the same binding mode as observed for class 1 with three consecutive ssDNA phosphate groups binding to the ps1β and h2i hairpins via identical residues. At the N-terminal tier, class 2 does not obviously differ from class 1 in the OB-fold and Zn-binding subdomains. These adopt an approximately 6-fold symmetric structure. Two of the peripheral helical subdomains of the N-terminal tier are poorly ordered for class 2 in contrast to the six well-ordered helical subdomains of class 1.

### 2.4. Mg/ATPase Sites

**Figure 4:**
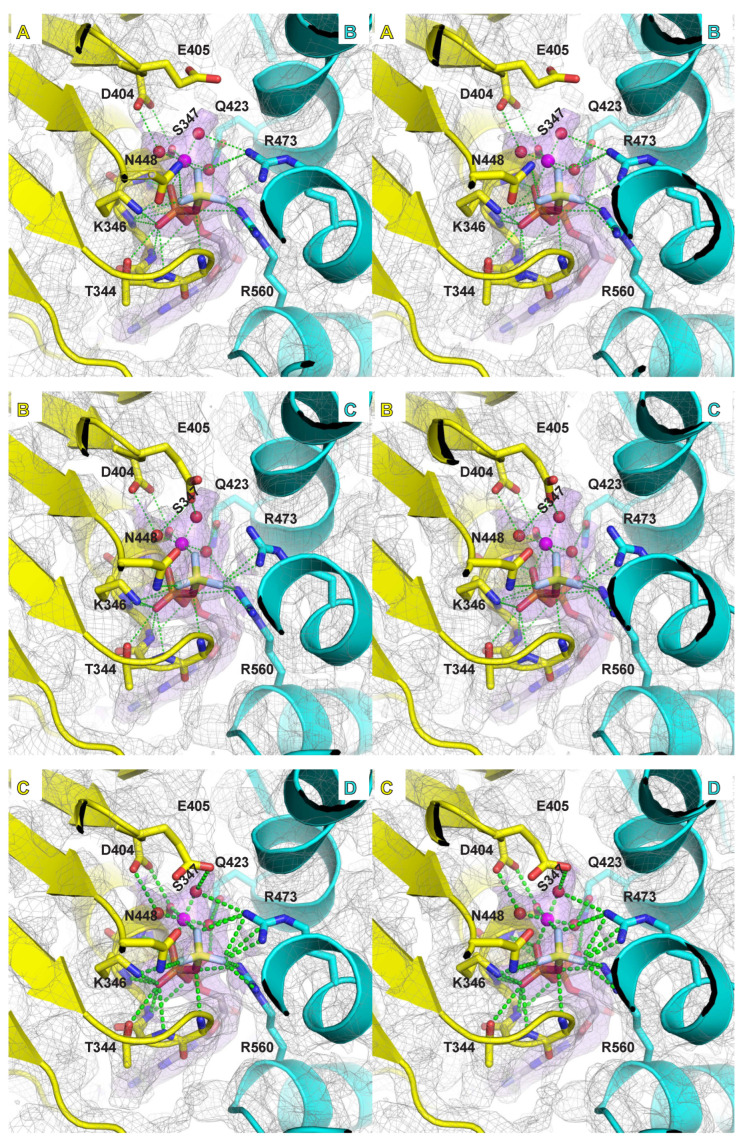

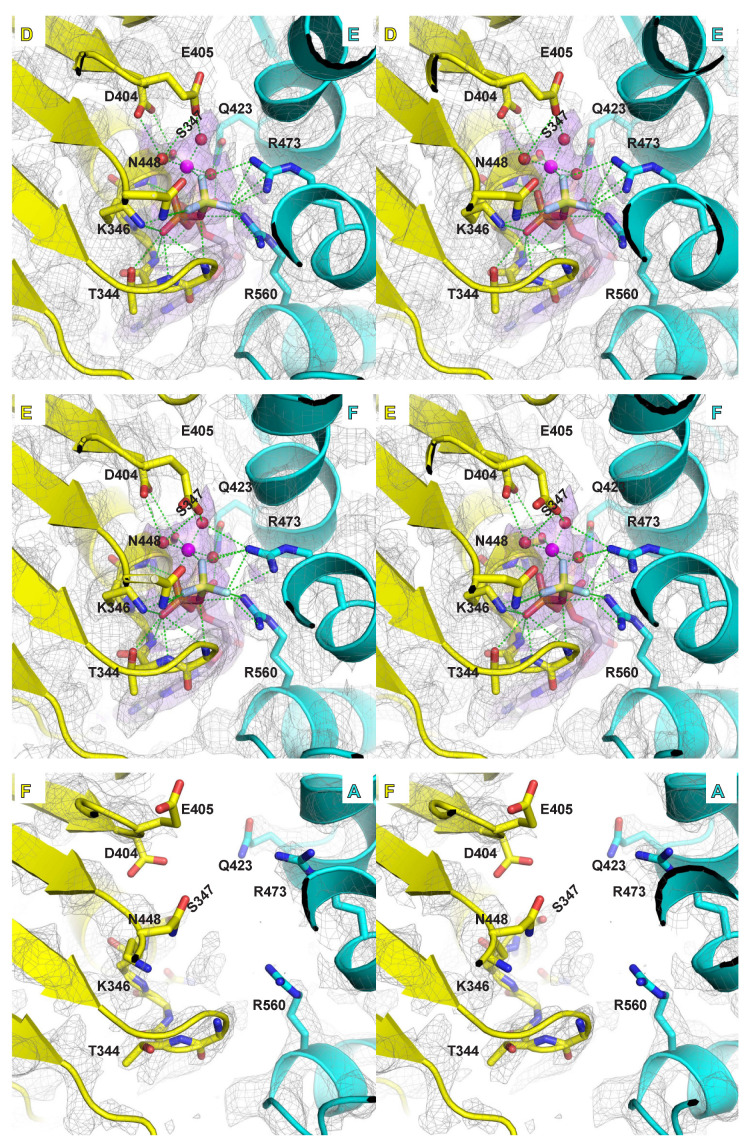
Stereoviews of the six subunit interfaces of class 1 illustrating the Mg/ATPase sites. The specific subunits comprising the interfaces are indicated by their chain identifiers (A,B,C,D,E,F). The illustrated maps and models were obtained by refinement of the merged particles of class 1 obtained for three different oligonucleotides (see Figures S10A, S11A and S12A). For each view, the subunit containing the Walker-A and -B residues is depicted in yellow, and the neighboring subunit with the arginine finger is depicted in cyan. Density above 5.0-sigma is shown in grey mesh with density within 2.5 Å of the modeled Mg/ATP also illustrated in purple transparent surface. Five interfaces show strong nucleotide density that do not strongly differ from each other. The sixth interface (F/A) is more open and does not show significant nucleotide density. Interactions involving Mg/ATP are depicted with green dashes. Interacting sidechains are shown in stick and labeled: T344, K346, and S347 of Walker-A; D404 and E405 of Walker-B; N448 of Sensor-1; R560 of Sensor-2; R473 arginine finger; and Q423. The magnesium ion in magenta has an octahedral coordination sphere derived from an oxygen of the β-phosphate, a fluoride of the BeF_3_ moiety, the conserved oxygen sidechain of S347, and three water molecules. Although functionally critical, the sidechain for Walker-B E405 is not well-ordered. Images were prepared with Pymol [48].

**Figure 5:**
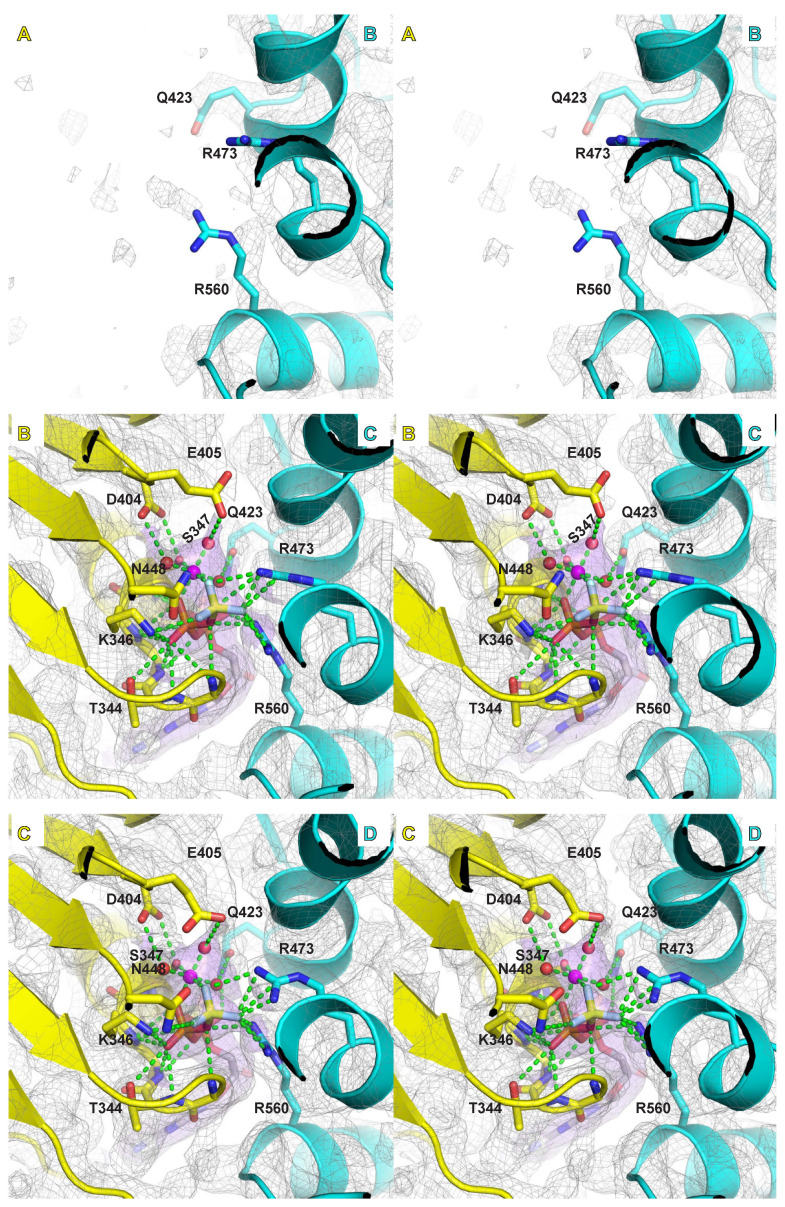

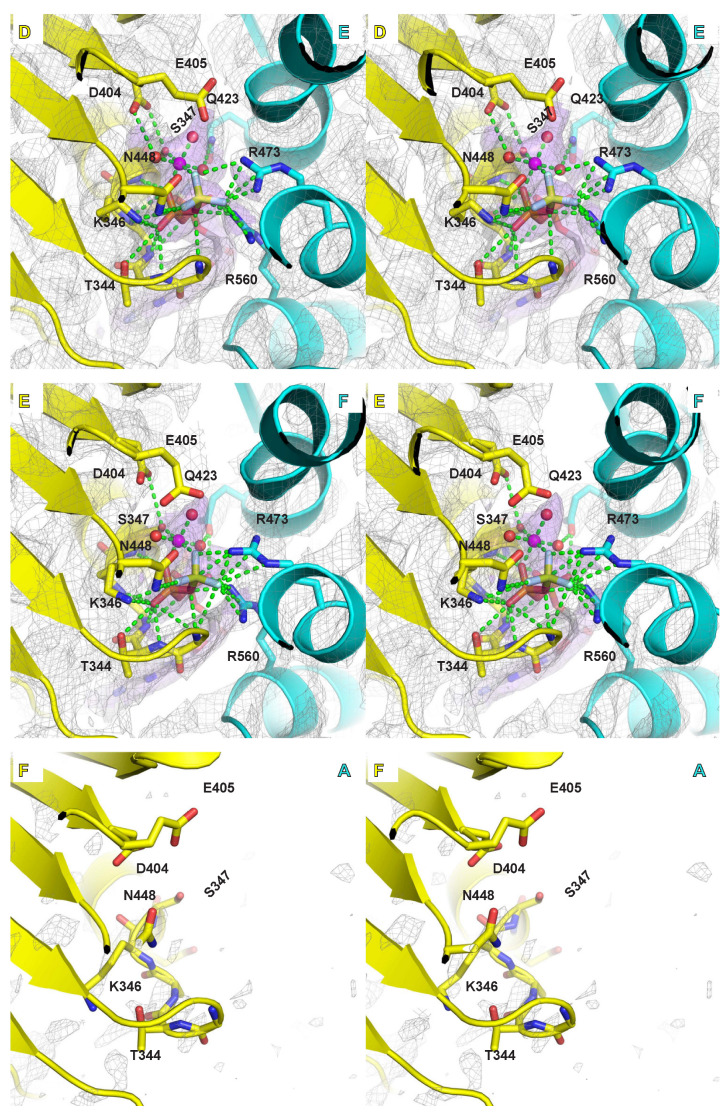
Stereoviews of the six subunit interfaces of class 2 illustrating the Mg/ATPase sites. The specific subunits comprising the interfaces are indicated by their chain identifiers (A,B,C,D,E,F). The illustrated maps and models were obtained by refinement of the merged particles of class 2 obtained for three different oligonucleotides (see Figures S10B, S11B and S12B). For each view, the subunit containing the Walker-A and -B residues is depicted in yellow, and the neighboring subunit with the arginine finger is depicted in cyan. Density above 5.0-sigma is shown in grey mesh with density within 2.5 Å of the modeled Mg/ATP also illustrated in purple transparent surface. Four interfaces show strong nucleotide density that do not strongly differ from each other. The ATPase domain of chain A is poorly ordered for class 2; hence, two of the interfaces (A/B and F/A) do not have a well-ordered Mg/ATP site. Interactions involving Mg/ATP are depicted with green dashes. Interacting sidechains are shown in stick and labeled: T344, K346, and S347 of Walker-A; D404 and E405 of Walker-B; N448 of Sensor-1; R560 of Sensor-2; R473 arginine finger; and Q423. The magnesium ion in magenta has an octahedral coordination sphere derived from an oxygen of the β-phosphate, a fluoride of the BeF_3_ moiety, the conserved oxygen sidechain of S347, and three water molecules. Although functionally critical, the sidechain for Walker-B E405 is not well-ordered. Images were prepared with Pymol [48].

Samples were prepared in the presence of magnesium and in situ generated ADP-BeF_3_, an ATP mimic that has proven highly useful for structural studies of ATPases in an ATP-bound form (see for example: [49,50]). In a previous crystal structure, we identified differences in the electron density among the MCM ATPase sites around the ring that we ascribed to different nucleotide states (ATP versus ADP). The higher resolution of the present structures indicates that all ATPase sites where a nucleotide is bound are highly similar in chemical structure (Figure 4, Figure 5 and Figures S10–S12), which we have assigned as ADP-BeF_3_. Although the general molecular shapes in the EM maps look highly similar for each of these ATPase sites, the strength of the density differs around the ring when proceeding from subunits that occupy the top of the staircase to those at the bottom. The differences in density strength among the sites around the ring could indicate different preferences for ATP versus ADP, analogous to the previous basis for assignment of ATPase site types based on crystallographic B-factors for the bacteriophage T7gp4 helicase [32].

The ATP binding and hydrolysis sites are formed at subunit interfaces with residues derived from two neighboring subunits. The specific residues that comprise the MCM ATPase site derive from sequence motifs that are characteristic of the AAA+ family of ATPases [10]. The principal amino acids that bind ATP and a magnesium ion are the Walker-A and Walker-B motif residues [51] of one subunit and the arginine-finger and sensor-II motif residues of the neighboring subunit (Figure 4, Figure 5 and Figures S10–S12). The higher resolution of the current structures allows more definitive assignments of the roles of these MCM residues. The nitrogens of six consecutive main-chain amides of the Walker-A motif (G343–Q348) bind to the α-phosphate and β-phosphate of groups of the ADP-BeF_3_. A conserved lysine of the Walker-A motif (K346) binds to a β-phosphate oxygen of the ADP-BeF_3_, and also to one fluoride of the BeF_3_ γ-phosphate mimic. The conserved serine/threonine of the Walker-A motif (S347) directly binds a magnesium ion. The magnesium ion also directly interacts with two atoms of the ADP-BeF_3_ molecule: an oxygen atom of the β-phosphate and one fluoride of the BeF_3_ γ-phosphate mimic. The magnesium ion also binds to three water molecules that each interact with MCM residues. Collectively, the six atoms that bind the magnesium ion provide an octahedral coordination sphere.

The Walker-B motif has two conserved consecutive acidic residues. The first, D404, forms a bidentate interaction with two of the atoms of the magnesium coordination sphere: the oxygen sidechain of the Walker-A conserved serine/threonine and a water molecule. This conserved residue is often mutated to asparagine in biochemical studies [52], and we predict that such a mutation would remain compatible with the coordination illustrated here. The second acidic residue, E405, is generally regarded as a catalytic base [53]. During catalysis, this residue activates a water molecule to attack the γ-phosphate of an ATP molecule, releasing an ADP molecule. This residue is frequently mutated in biochemical studies to ablate ATP hydrolysis. Despite the critical role of this residue in ATP hydrolysis, the sidechain of this residue is weakly ordered in our structures compared to other parts of the ATPase site. As a result, this sidechain does not visibly form significant interactions with neighboring residues, the nucleotide, or the magnesium coordination sphere.

The neighboring subunit of the ATPase site principally contributes two conserved arginine residues, the “arginine finger” and an arginine from the sensor-II motif. The “arginine finger”, sometimes referred to as the “SRF motif” in MCM proteins [20], is essential for ATP hydrolysis and was previously used to determine the order of subunits around the hexameric ring for eukaryotic Mcm2-7 [20]. In our high-resolution structure, the arginine finger, R473, generally forms a bidentate interaction with two fluorides of the BeF_3_ γ-phosphate mimic. The arginine of the sensor-II motif, R560, interacts with the oxygen bridging the β-phosphate to the BeF_3_ group, one fluoride of the BeF_3_ group, and an oxygen of the α-phosphate. Hence, the sensor-II arginine forms interactions with all three phosphates when binding ATP. At some interfaces, this arginine interacts with a water molecule of the magnesium coordination sphere. It also interacts with the hydroxyl sidechain of the conserved serine of the arginine finger SRF motif. The second subunit of the ATPase site also contributes a conserved glutamine (Q498; see “Q” in Figure S5) that interacts with a water molecule of the magnesium coordination sphere and the α-phosphate of the ATP mimic.

The above Interactions orient and activate the triphosphate group of ATP for hydrolysis. Additional interactions with the adenine ring and the sugar group help increase binding affinity for ATP. The main-chain amide of Y304 (Y304 N and Y304 O) from one subunit interacts with the adenine ring. The sidechain of E563 of the neighboring subunit forms a bidentate interaction with two oxygens of the sugar.

## 3. Discussion

### Translocation Mechanism

The binding of ssDNA to hexameric helicases in a spiral staircase, which has thus far been universal for hexameric helicases [5,7,22,34,35,36], provides a straightforward two-step ATPase-driven mechanism for translocation. In this mechanism, the collective staircase descends upon ATP-hydrolysis, and then the subunit at the bottom of the staircase moves to the top of the staircase upon binding ATP [5]. This mechanism calls for dissociation and re-association of one MCM subunit and the DNA such that a different number of subunits engage in binding DNA at different times. The two classes of structure presented here illustrate two different numbers of subunits engaged to DNA and allow us to more thoroughly define a “hand-over-hand” mechanism used by the MCM ring to translocate ssDNA.

We suggest that the two classes reported here illustrate the molecular basis for one subunit’s dissociation/re-association during DNA translocation. Specifically, transformation of class 1 with six subunits engaged to DNA to class 2 with five subunits engaged illustrates a dissociation of one subunit from DNA. Similarly, the transformation of class 2 to class 1 illustrates re-association of one subunit with DNA. Sequential permutation of these two transformations around the ring creates a sequential rotary cycle that translocates the strand of DNA through the ring channel (Supplementary Movies S1 and S2). Notably, the two unique hexamers of the crystal structure of papillomavirus E1 helicase bound to ssDNA also showed a different number of subunits bound to ssDNA [5], suggesting a potentially universal mechanism of DNA translocation by hexameric helicases with oscillating dissociation and re-association events.

A surprising aspect of the structures is the highly disordered ATPase domain of class 2. This domain is not tethered to its neighboring subunits or to the DNA, and on this basis would be expected to be the most mobile of the ATPase domains, but it is surprising to find it so highly disordered. The extreme mobility of this domain in this class may be functionally important to facilitate the hand-over-hand mechanism. In the sequential rotary mechanism that we illustrated for *Sso*MCM based on our crystal structure [36], the transition of the subunit at the bottom of the staircase to the top of the staircase appears difficult. During this transition, the bottom hairpins need to get on top of the strand of DNA immediately upstream of the first hairpin of the staircase. If the subunit moves in a direct, linear path defined by the six well-ordered subunits of our crystal structure [36], the two DNA-binding hairpins could collide with the DNA strand rather than move above it. Further, this region of the DNA strand is anticipated to be under tension due to the downward force exerted by the helicase, making it inflexible to allow bypass of the leapfrogging hairpin. The disordered ATPase subunit of class 2 suggests that this subunit could significantly pull away from the complex, increasing its distance from the DNA and enabling it to move above the DNA strand before re-engaging its binding to DNA. The extreme mobility of the ATPase domain in this class could also be functionally important for releasing ADP from the associated ATPase site to prime it for binding a new ATP molecule.

**Figure 6:**
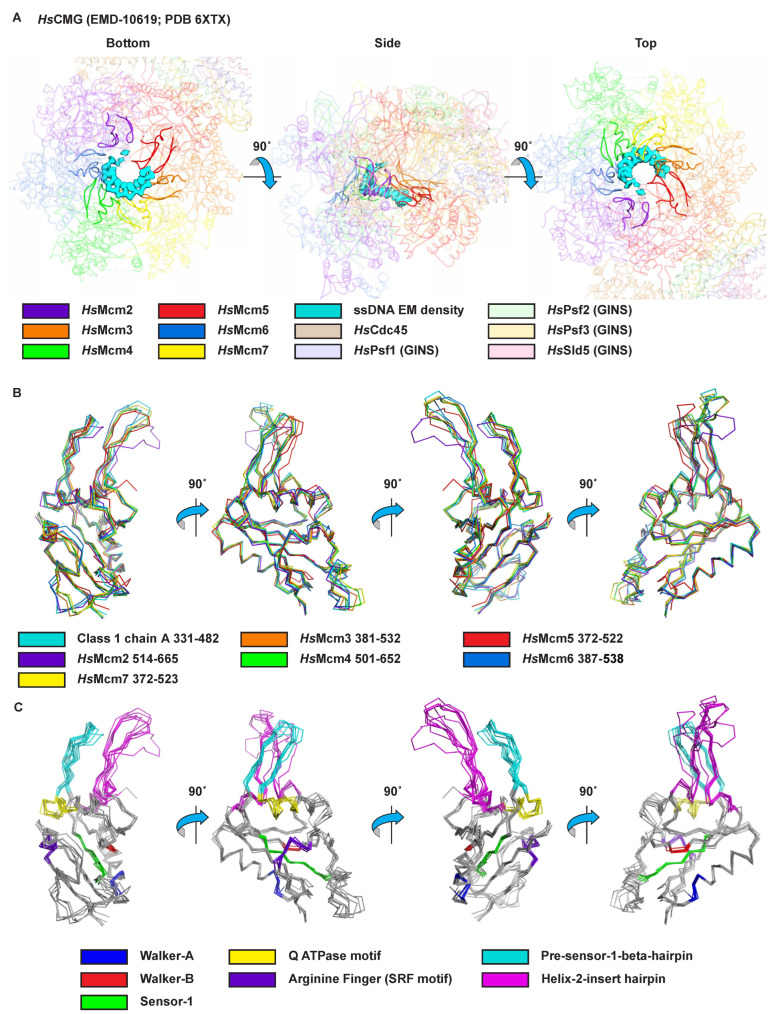
The core MCM ATPase domains of eukaryotes are archaea are highly similar in structure and function. (**A**) The human CMG complex binds DNA with a spiral staircase of hairpins (PDB 6XTX and EMD-10619 [22]) analogous to that of *Sso*MCM. Three perpendicular views are provided analogous to those of Figure 3A, illustrating highly similar ssDNA density and binding mode with the two hairpins of each subunit. The “side” view is perpendicular to the central channel with the N-terminal tier on top and the ATPase tier on the bottom. The “bottom” view is parallel to the central channel from the ATPase tier side. The “top” view is parallel to the central channel from the N-terminal tier side. (**B**) The 152-amino-acid core ATPase domain structures are superimposed and colored by subunit. The core structure is highly conserved in structure with the most prominent variability in the helix-2-insert hairpins of *Hs*Mcm2 and *Hs*Mcm5, which occupy the “top” and “bottom” positions of the staircase (see (**A**)) and are thus anticipated to be more mobile. (**C**) The superimposed structures are colored according to their DNA-binding and ATPase motifs (see Figure S5). See also Movie S3. (**A**,**B**) were prepared with Chimera [47], and (**C**) was prepared with Pymol [48].

All MCM AAA+ ATPase domains are extremely similar in structure with fully conserved residues for DNA-binding and ATPase activities. With rare and extremely minor exceptions, the core structure is an invariantly structured contiguous stretch of 152 amino acids without insertion or deletion (Figure 2 and Figure S5). This invariant sequence length includes all core β-strands of the AAA+ fold, the Walker-A, Walker-B, and arginine finger ATPase motifs, and the helix-2-insert and pre-sensor-1-β DNA-binding hairpins. Hence, the core portion of all MCM hexameric rings that fulfills ATPase and DNA-binding activities is highly symmetric even though the subunits may differ around the ring. The core of the ATPase domain is also strongly conserved in structure as illustrated by comparison with the structures of the six ATPase domains in the high-resolution cryo-EM structure of the human CMG complex bound to DNA (PDB 6XTX and EMD-10619 [22], Figure 6 and Movie S3). Further, the ATPase tier of the human heterohexamer binds DNA indistinguishably from the homohexamer of *Sso*MCM (Compare Figure 6A to Figure 3A). Other eukaryotic MCM heterohexamers show modes of binding to DNA that do not appear to differ from the structures of Figure 3A and Figure 6A at the resolutions obtained [54,55,56].

The fully conserved structure, ATPase residues, and DNA-binding residues in this core module at each position around the ring indicates that all attributes of a sequential rotary ssDNA translocation mechanism as described above for the archaeal MCM homohexamer are also fully possible for the heterohexameric eukaryotic Mcm2-7 ring. Such a mechanism would involve equal participation of each ATPase site. However, ATPase site mutants do not show equivalent defects of unwinding for Drosophila CMG [54,57] and biochemical unwinding shows uniquely strong dependence on select ATPase sites, particularly Mcm3 and Mcm5, leading to the suggestion that eukaryotic CMG uses an asymmetric mechanism to pull one strand of DNA through its central pore [54] that differs from the sequential, rotary mechanism believed to operate for all other hexameric ring helicases. An alternative interpretation that is more unifying in fundamental ring helicase mechanism is that the Mcm3 and Mcm5 ATPase sites could have a specialized role that is necessary to first initiate DNA unwinding (in cells and biochemically), and that once initiated, all subunits could participate in a sequential, rotary mechanism analogous to those of homohexameric rings.

## 4. Materials and Methods

### 4.1. Protein Expression and Purification

An N-terminal His_6_-SUMO-fusion of the MCM protein of the archaeal organism *Saccharolobus solfataricus* (*Sso*MCM) with the C-terminal helix-turn-helix domain removed and a modified linker between the N-terminal domain and the AAA+ ATPase domain was expressed in BL21(DE3)-RIPL cells and purified as described previously (pEE078.1 [36]). The specific interdomain linker modification replaced nine amino acids (266–274) acids with the six residue GGSGGS sequence. Briefly, the protein was expressed in LB media supplemented with 0.4% glucose and 30 mg/L kanamycin. Harvested cells were lysed, clarified by centrifugation, and cellular nucleic acids were precipitated with 0.3% final concentration polyethylenimine. The supernatant was subjected to ammonium sulfate precipitation (70% saturation), and the pellet was isolated by centrifugation. The pellet was resuspended and purified by Ni-NTA chromatography followed by size-exclusion chromatography. The sample was dialyzed overnight in the presence of Ulp1 protease to remove the SUMO tag (the Ulp1 protease plasmid was the generous gift of Dr. Christopher D. Lima [58] under MTA). The MCM protein was then subjected to anion exchange followed by size-exclusion chromatography. A single homogenous peak at a volume consistent with a hexamer was highly pure based on SDS-PAGE. These fractions were pooled and concentrated in a spin concentrator.

### 4.2. Cryo-EM Sample Preparation

The protein sample was maintained at a concentration of 1 mg/mL. Prior to freezing, the sample was mixed 5:1 by volume with 1 mM oligonucleotide, and freshly prepared ADP-BeF_3_ was added to 5 mM final concentration. The resulting sample was applied to a grid and plunge-frozen in liquid ethane with a Vitrobot. Three different oligonucleotides were used to generate three distinct samples and cryo-EM datasets. The specific oligonucleotides consisted of 16-mer poly-dT, 12-mer poly-dT, and a TTTTTTTTTTTTTTTTTTTTCTATAGTTTTTTTTTTTTTTTTTTTT sequence, which has potential to form an “X-shape” with 20-mer poly-dT arms based on the central 6-nucleotide palindromic sequence.

### 4.3. Data Collection, Structure Determination, and Structure Refinement

Cryo-EM data were collected using Titan Krios (ThermoFisher, Waltham, MA, USA) transmission electron microscope, equipped with a K3 direct electron detector and post-column GIF (energy filter). K3 dark and gain reference were collected just before data collection. Data collection was performed in SerialEM software (SerialEM_3-5-9) [59] with image shift protocol (9 images were collected with one defocus measurements). Movies were recorded at defocus values from −0.8 to −1.8 µm at a magnification of 81kx, which corresponds to the pixel size of 1.08 Å at the specimen level (super resolution pixel size is 0.54). During 3-s exposure, 60 frames (0.05 s per frame and the dose of 1.3 e^−^/frame/Å^2^) were collected with the total dose of ~78 e^−^. Motion correction was performed on raw super resolution movie stacks and binned by 2 using MotionCor2 software (MotionCor2_1.2.1-Cuda92) [60]. Initial CTF parameters were determined with Patch CTF in cryoSPARC (version 3.3.1) [61]. Particles were picked in cryoSPARC [61]. Several rounds of 2D classification were performed to eliminate ice, carbon edges, and false-positive particles containing noise. Details of the individual sample refinements are provided below. In order to further improve the structures, CTF-refinements were performed prior to final homogeneous refinement in cryoSPARC. The sharpened density maps from cryoSPARC homogeneous refinement were used to produce figures.

### 4.4. SsoMCM–T16–MgADP-BeF_3_

A total of 5171 images were collected. A set of 38,306 particles were picked from 20 micrographs with Blob picker of cryoSPARC [61]. These were extracted and subjected to two rounds of 2D classification. The selected classes were used as templates in the Template picker tool of cryoSPARC to select 10,298,489 particles from the full set of micrographs. After 3 total rounds of 2D-classification in Relion (RELION 3.0.7) [62] and cryoSPARC and duplicate removal, 2,839,559 particles remained in the set. An ab initio structure was constructed in cryoSPARC in C_1_-symmetry, which was then used as the reference map for homogeneous refinement in cryoSPARC [61] with C_1_-symmetry. The refined structure appeared 6-fold symmetric, including a segment of ssDNA in the central channel that looked like a closed, roughly planar circle. This structure had an apparent resolution of 3.0 Å based on GSFSC. This structure was then homogeneously refined with cryoSPARC in C_6_-symmetry to align its pseudo-C_6_ axis with the coordinate system. The particles were then 6-fold expanded about the C_6_-axis in Relion [62]. The expanded particle set was subjected to 3D-classification with local refinement in Relion [62] to generate six classes. This classification used a masked region comprising the central channel hairpins of the MCM ATPase tier and the bound ssDNA. The resulting six classes showed six permutations of a helical segment of ssDNA bound to a spiral staircase of hairpins on the protein ring. One of these permutations was then used as the reference map in homogeneous cryoSPARC refinement with the original unexpanded particle set. The resulting map was used as the initial reference map in the structures with other oligonucleotides (described below) and was used as the reference map in cryoSPARC heterogeneous refinement in two classes. Convergence of the heterogeneous refinement required 50 iterations of the full particle set. The especially large number of iterations required is likely due to the near 6-fold symmetry of a large portion of the structural forms and their similarity to each other. Further heterogeneous refinement in cryoSPARC of the smaller class indicated a minor fraction of partial ring particles that were excluded from further consideration. Each of the two classes were subjected to homogeneous refinement in cryoSPARC, including CTF refinement. The final refinement of class 1 included 1,649,356 particles and provided a structure with a GSFCSC of 2.48 Å (Figure S1). The final refinement of class 2 included 776,823 particles and a provided a structure with a GSFCSC of 2.76 Å (Figure S1).

### 4.5. SsoMCM–T20-CTATAG-T20–MgADP-BeF_3_

A total of 2718 images were collected. A set of 39,559 particles were picked from 20 micrographs with Blob picker of cryoSPARC [61]. These were extracted and subjected to two rounds of 2D classification. The selected classes were used as templates in the Template picker tool of cryoSPARC [61] to select 3,794,897 particles from the full set of micrographs. After 4 total rounds of 2D-classification in cryoSPARC [61] and duplicate removal, 1,568,736 particles remained in the set. The spiral staircase model of *Sso*MCM–T16–MgADP-BeF_3_ obtained after initial cryoSPARC homogeneous refinement of the full particle set was used as the reference map to initiate 50 iterations of cryoSPARC heterogeneous refinement in two classes. The refinement behaved similar to that of *Sso*MCM–T16–MgADP-BeF_3_. Heterogeneous refinement in cryoSPARC of the smaller class indicated a minor fraction of partial ring particles that were excluded from further consideration. Each of the two classes were subjected to homogeneous refinement in cryoSPARC, including CTF refinement. The final refinement of class 1 included 832,313 particles and provided a structure with GSFCSC of 2.45 Å (see Figure S1). The final refinement of class 2 included 448,473 particles and provided a structure with GSFCSC of 2.67 Å (Figure S1).

### 4.6. SsoMCM–T12–MgADP-BeF_3_

A total of 1928 images were collected. A set of 567,023 particles were picked from 500 micrographs with Blob picker of cryoSPARC [61]. These were extracted and subjected to one round of 2D classification. The selected classes were used as templates in the Template picker tool of cryoSPARC to select 265,202 particles from the 500 micrographs. These were subjected to one round of 2D classification, and the selected classes were used as templates in the Template picker tool of cryoSPARC to select 1,677,930 particles from the full set of micrographs. After 3 rounds of 2D-classification in cryoSPARC and duplicate removal, 1,067,534 particles remained in the set. The spiral staircase model of SsoMCM–T16–MgADP-BeF_3_ obtained after initial cryoSPARC homogeneous refinement of the full particle set was used as the reference map to initiate 50 iterations of cryoSPARC heterogeneous refinement in two classes. The refinement behaved similar to that of SsoMCM–T16–MgADP-BeF_3_. Heterogeneous refinement in cryoSPARC of the smaller class indicated a minor fraction of partial ring particles that were excluded from further consideration. Each of the two classes were subjected to homogeneous refinement in cryoSPARC, including CTF refinement. The final refinement of class 1 included 645,002 particles and a GSFCSC of 2.62 Å (Figure S1). The final refinement of class 2 included 268,300 particles and a GSFCSC of 3.01 Å (Figure S1).

### 4.7. Merged Structures

The particles for the two classes were pooled respectively and subjected to homogeneous refinement in cryoSPARC. The final refinement of class 1 included 3,126,671 particles and a GSFCSC of 2.34 Å (Figure S1). The final refinement of class 2 included 1,493,596 particles and a GSFCSC of 2.59 Å (Figure S1).

## Figures and Tables

**Table 1:** Cryo-EM statistics.

Dataset	*Sso*MCM–T12– MgADP-BeF_3_		*Sso*MCM–T16– MgADP-BeF_3_		*Sso*MCM– T20-CTATAG-T20– MgADP-BeF_3_		Merged Particles	
Microscope	Titan Krios		Titan Krios		Titan Krios		N/A	
kEV	300		300		300		N/A	
Micrographs	1928		5171		2718		N/A	
Detector	K3		K3		K3		N/A	
Magnification	81,000		81,000		81,000		N/A	
Pixel size (Å)	1.08		1.08		1.08		N/A	
Particles after 2D classification	1,067,534		2,839,559		1,568,736		N/A	
Class	1	2	1	2	1	2	1	2
Particles	645,002	268,300	1,649,356	776,823	832,313	448,473	3,126,671	1,493,596
Resolution * (Å)	2.62	3.01	2.48	2.76	2.45	2.67	2.34	2.59

* GSFC = 0.143.

## Data Availability

The structures have been deposited at the PDB with the following accession codes:
*Sso*MCM–T12–MgADP-BeF_3_ class 1:8EAF, EMD-27974*Sso*MCM–T12–MgADP-BeF_3_ class 2:8EAG, EMD-27975*Sso*MCM–T16–MgADP-BeF_3_ class 1:8EAH, EMD-27976*Sso*MCM–T16–MgADP-BeF_3_ class 2:8EAI, EMD-27977*Sso*MCM–T20-CTATAG-T20–MgADP-BeF_3_ class 1:8EAJ, EMD-27978*Sso*MCM–T20-CTATAG-T20–MgADP-BeF_3_ class 2:8EAK, EMD-27979Merged class 1 particles:8EAL, EMD-27980Merged class 2 particles:8EAM, EMD-27981

## Supplementary Materials

The following supporting information can be downloaded at: https://www.mdpi.com/article/10.3390/ijms232314678/s1.

## References

[1] Bell S.P., Labib K. (2016). Chromosome Duplication in Saccharomyces cerevisiae. Genetics.

[2] Patel S.S., Picha K.M. (2000). Structure and function of hexameric helicases. Annu. Rev. Biochem..

[3] Gao Y., Yang W. (2020). Different mechanisms for translocation by monomeric and hexameric helicases. Curr. Opin. Struct. Biol..

[4] Yu X., Hingorani M.M., Patel S.S., Egelman E.H. (1996). DNA is bound within the central hole to one or two of the six subunits of the T7 DNA helicase. Nat. Struct. Biol..

[5] Enemark E.J., Joshua-Tor L. (2006). Mechanism of DNA translocation in a replicative hexameric helicase. Nature.

[6] Fu Y.V., Yardimci H., Long D.T., Ho T.V., Guainazzi A., Bermudez V.P., Hurwitz J., van Oijen A., Scharer O.D., Walter J.C. (2011). Selective bypass of a lagging strand roadblock by the eukaryotic replicative DNA helicase. Cell.

[7] Itsathitphaisarn O., Wing R.A., Eliason W.K., Wang J., Steitz T.A. (2012). The Hexameric Helicase DnaB Adopts a Nonplanar Conformation during Translocation. Cell.

[8] Lee S.J., Syed S., Enemark E.J., Schuck S., Stenlund A., Ha T., Joshua-Tor L. (2014). Dynamic look at DNA unwinding by a replicative helicase. Proc. Natl. Acad. Sci. USA.

[9] Singleton M.R., Dillingham M.S., Wigley D.B. (2007). Structure and mechanism of helicases and nucleic acid translocases. Annu. Rev. Biochem..

[10] Neuwald A.F., Aravind L., Spouge J.L., Koonin E.V. (1999). AAA+: A class of chaperone-like ATPases associated with the assembly, operation, and disassembly of protein complexes. Genome Res..

[11] Chong J.P., Hayashi M.K., Simon M.N., Xu R.M., Stillman B. (2000). A double-hexamer archaeal minichromosome maintenance protein is an ATP-dependent DNA helicase. Proc. Natl. Acad. Sci. USA.

[12] Gorbalenya A.E., Koonin E.V., Wolf Y.I. (1990). A new superfamily of putative NTP-binding domains encoded by genomes of small DNA and RNA viruses. FEBS Lett..

[13] Simmons D.T. (2000). SV40 large T antigen functions in DNA replication and transformation. Adv. Virus Res..

[14] Stenlund A. (2003). Initiation of DNA replication: Lessons from viral initiator proteins. Nat. Rev. Mol. Cell Biol..

[15] Oakley A.J. (2019). A structural view of bacterial DNA replication. Protein Sci..

[16] Peter B., Falkenberg M. (2020). TWINKLE and Other Human Mitochondrial DNA Helicases: Structure, Function and Disease. Genes.

[17] Kulczyk A.W., Richardson C.C. (2016). The Replication System of Bacteriophage T7. Enzymes.

[18] Brennan C.A., Dombroski A.J., Platt T. (1987). Transcription termination factor rho is an RNA-DNA helicase. Cell.

[19] Tye B.K. (1999). MCM proteins in DNA replication. Annu. Rev. Biochem..

[20] Davey M.J., Indiani C., O’Donnell M. (2003). Reconstitution of the Mcm2-7p heterohexamer, subunit arrangement, and ATP site architecture. J. Biol. Chem..

[21] Li N., Zhai Y., Zhang Y., Li W., Yang M., Lei J., Tye B.K., Gao N. (2015). Structure of the eukaryotic MCM complex at 3.8 A. Nature.

[22] Rzechorzek N.J., Hardwick S.W., Jatikusumo V.A., Chirgadze D.Y., Pellegrini L. (2020). CryoEM structures of human CMG-ATPgammaS-DNA and CMG-AND-1 complexes. Nucleic Acids Res..

[23] Moyer S.E., Lewis P.W., Botchan M.R. (2006). Isolation of the Cdc45/Mcm2-7/GINS (CMG) complex, a candidate for the eukaryotic DNA replication fork helicase. Proc. Natl. Acad. Sci. USA.

[24] Xu Y., Gristwood T., Hodgson B., Trinidad J.C., Albers S.V., Bell S.D. (2016). Archaeal orthologs of Cdc45 and GINS form a stable complex that stimulates the helicase activity of MCM. Proc. Natl. Acad. Sci. USA.

[25] Yeeles J.T., Deegan T.D., Janska A., Early A., Diffley J.F. (2015). Regulated eukaryotic DNA replication origin firing with purified proteins. Nature.

[26] Bochman M.L., Schwacha A. (2008). The Mcm2-7 complex has in vitro helicase activity. Mol. Cell.

[27] Boyer P.D. (1997). The ATP synthase—A splendid molecular machine. Annu. Rev. Biochem..

[28] Boyer P.D. (1993). The binding change mechanism for ATP synthase—Some probabilities and possibilities. Biochim. Biophys. Acta.

[29] Stock D., Gibbons C., Arechaga I., Leslie A.G., Walker J.E. (2000). The rotary mechanism of ATP synthase. Curr. Opin. Struct. Biol..

[30] Sobti M., Ueno H., Noji H., Stewart A.G. (2021). The six steps of the complete F1-ATPase rotary catalytic cycle. Nat. Commun..

[31] Egelman E.H., Yu X., Wild R., Hingorani M.M., Patel S.S. (1995). Bacteriophage T7 helicase/primase proteins form rings around single-stranded DNA that suggest a general structure for hexameric helicases. Proc. Natl. Acad. Sci. USA.

[32] Singleton M.R., Sawaya M.R., Ellenberger T., Wigley D.B. (2000). Crystal structure of T7 gene 4 ring helicase indicates a mechanism for sequential hydrolysis of nucleotides. Cell.

[33] Abrahams J.P., Leslie A.G., Lutter R., Walker J.E. (1994). Structure at 2.8 A resolution of F1-ATPase from bovine heart mitochondria. Nature.

[34] Gao Y., Cui Y., Fox T., Lin S., Wang H., de Val N., Zhou Z.H., Yang W. (2019). Structures and operating principles of the replisome. Science.

[35] Thomsen N.D., Berger J.M. (2009). Running in reverse: The structural basis for translocation polarity in hexameric helicases. Cell.

[36] Meagher M., Epling L.B., Enemark E.J. (2019). DNA translocation mechanism of the MCM complex and implications for replication initiation. Nat. Commun..

[37] Pape T., Meka H., Chen S., Vicentini G., van Heel M., Onesti S. (2003). Hexameric ring structure of the full-length archaeal MCM protein complex. EMBO Rep..

[38] Costa A., Pape T., van Heel M., Brick P., Patwardhan A., Onesti S. (2006). Structural basis of the Methanothermobacter thermautotrophicus MCM helicase activity. Nucleic Acids Res..

[39] Bochman M.L., Schwacha A. (2007). Differences in the single-stranded DNA binding activities of MCM2-7 and MCM467: MCM2 and MCM5 define a slow ATP-dependent step. J. Biol. Chem..

[40] Remus D., Beuron F., Tolun G., Griffith J.D., Morris E.P., Diffley J.F. (2009). Concerted loading of Mcm2-7 double hexamers around DNA during DNA replication origin licensing. Cell.

[41] Costa A., Ilves I., Tamberg N., Petojevic T., Nogales E., Botchan M.R., Berger J.M. (2011). The structural basis for MCM2-7 helicase activation by GINS and Cdc45. Nat. Struct. Mol. Biol..

[42] Yuan Z., Bai L., Sun J., Georgescu R., Liu J., O’Donnell M.E., Li H. (2016). Structure of the eukaryotic replicative CMG helicase suggests a pumpjack motion for translocation. Nat. Struct. Mol. Biol..

[43] Noguchi Y., Yuan Z., Bai L., Schneider S., Zhao G., Stillman B., Speck C., Li H. (2017). Cryo-EM structure of Mcm2-7 double hexamer on DNA suggests a lagging-strand DNA extrusion model. Proc. Natl. Acad. Sci. USA.

[44] Fletcher R.J., Bishop B.E., Leon R.P., Sclafani R.A., Ogata C.M., Chen X.S. (2003). The structure and function of MCM from archaeal *M. Thermoautotrophicum*. Nat. Struct. Biol..

[45] Miller J.M., Enemark E.J. (2015). Archaeal MCM Proteins as an Analog for the Eukaryotic Mcm2-7 Helicase to Reveal Essential Features of Structure and Function. Archaea.

[46] Croll T.I. (2018). ISOLDE: A physically realistic environment for model building into low-resolution electron-density maps. Acta Crystallogr. D Struct. Biol..

[47] Pettersen E.F., Goddard T.D., Huang C.C., Couch G.S., Greenblatt D.M., Meng E.C., Ferrin T.E. (2004). UCSF Chimera—A visualization system for exploratory research and analysis. J. Comput. Chem..

[48] (2010). The PyMOL Molecular Graphics System.

[49] Kagawa R., Montgomery M.G., Braig K., Leslie A.G., Walker J.E. (2004). The structure of bovine F1-ATPase inhibited by ADP and beryllium fluoride. EMBO J..

[50] Gai D., Zhao R., Li D., Finkielstein C.V., Chen X.S. (2004). Mechanisms of conformational change for a replicative hexameric helicase of SV40 large tumor antigen. Cell.

[51] Walker J.E., Saraste M., Runswick M.J., Gay N.J. (1982). Distantly related sequences in the alpha- and beta-subunits of ATP synthase, myosin, kinases and other ATP-requiring enzymes and a common nucleotide binding fold. EMBO J..

[52] Bochman M.L., Bell S.P., Schwacha A. (2008). Subunit organization of Mcm2-7 and the unequal role of active sites in ATP hydrolysis and viability. Mol. Cell Biol..

[53] Leipe D.D., Koonin E.V., Aravind L. (2003). Evolution and classification of P-loop kinases and related proteins. J. Mol. Biol..

[54] Eickhoff P., Kose H.B., Martino F., Petojevic T., Abid Ali F., Locke J., Tamberg N., Nans A., Berger J.M., Botchan M.R. (2019). Molecular Basis for ATP-Hydrolysis-Driven DNA Translocation by the CMG Helicase of the Eukaryotic Replisome. Cell Rep..

[55] Yuan Z., Georgescu R., Bai L., Zhang D., Li H., O’Donnell M.E. (2020). DNA unwinding mechanism of a eukaryotic replicative CMG helicase. Nat. Commun..

[56] Baretic D., Jenkyn-Bedford M., Aria V., Cannone G., Skehel M., Yeeles J.T.P. (2020). Cryo-EM Structure of the Fork Protection Complex Bound to CMG at a Replication Fork. Mol. Cell.

[57] Ilves I., Petojevic T., Pesavento J.J., Botchan M.R. (2010). Activation of the MCM2-7 helicase by association with Cdc45 and GINS proteins. Mol. Cell.

[58] Mossessova E., Lima C.D. (2000). Ulp1-SUMO crystal structure and genetic analysis reveal conserved interactions and a regulatory element essential for cell growth in yeast. Mol. Cell.

[59] Mastronarde D.N. (2005). Automated electron microscope tomography using robust prediction of specimen movements. J. Struct. Biol..

[60] Zheng S.Q., Palovcak E., Armache J.P., Verba K.A., Cheng Y., Agard D.A. (2017). MotionCor2: Anisotropic correction of beam-induced motion for improved cryo-electron microscopy. Nat. Methods.

[61] Punjani A., Rubinstein J.L., Fleet D.J., Brubaker M.A. (2017). cryoSPARC: Algorithms for rapid unsupervised cryo-EM structure determination. Nat. Methods.

[62] Zivanov J., Nakane T., Forsberg B.O., Kimanius D., Hagen W.J., Lindahl E., Scheres S.H. (2018). New tools for automated high-resolution cryo-EM structure determination in RELION-3. eLife.

