# Supplementary material for: Two Distinct Modes of DNA Binding by an MCM Helicase Enable DNA Translocation
Source: Int J Mol Sci. 2022 Nov 24;23(23):14678. doi: 10.3390/ijms232314678 (PMC9735655; doi:10.3390/ijms232314678)
Supplement: Supplementary file 1 [file ijms-23-14678-s001.zip › Supplementary-112322.pdf]

Two distinct modes of DNA binding by an MCM helicase enable DNA translocation

Supplementary information

Martin Meagher[1,†], Alexander Myasnikov[1,†], Eric J. Enemark[1,2*]

[1]Department of Structural Biology, St Jude Children's Research Hospital, 262 Danny Thomas Place, Mail Stop 311, Memphis, TN 38105, USA

[2]Department of Biochemistry and Molecular Biology, University of Arkansas for Medical Sciences, 4301 W. Markham St., slot 516, Little Rock, AR 72205, USA

[†]These authors contributed equally to this work.
To whom correspondence should be addressed: ejenemark@uams.edu

Present Address:
Dr. Martin Meagher, Tome Biosciences, 100 Talcott Ave, Watertown, MA 02472

Present Address:
Dr. Alexander Myasnikov
Head of Dubochet Center for Imaging Lausanne (DCI-Lausanne) at EPFL
EPFL VPA DCI-Lausanne
BSP 407 / Bâtiment Cubotron
Route de Sorge
CH-1015 Lausanne, Switzerland
Email: alexander.myasnikov@epfl.ch
Mobile: +41 764496664
Office: +41 216930444
web: https://www.dci-lausanne.org/

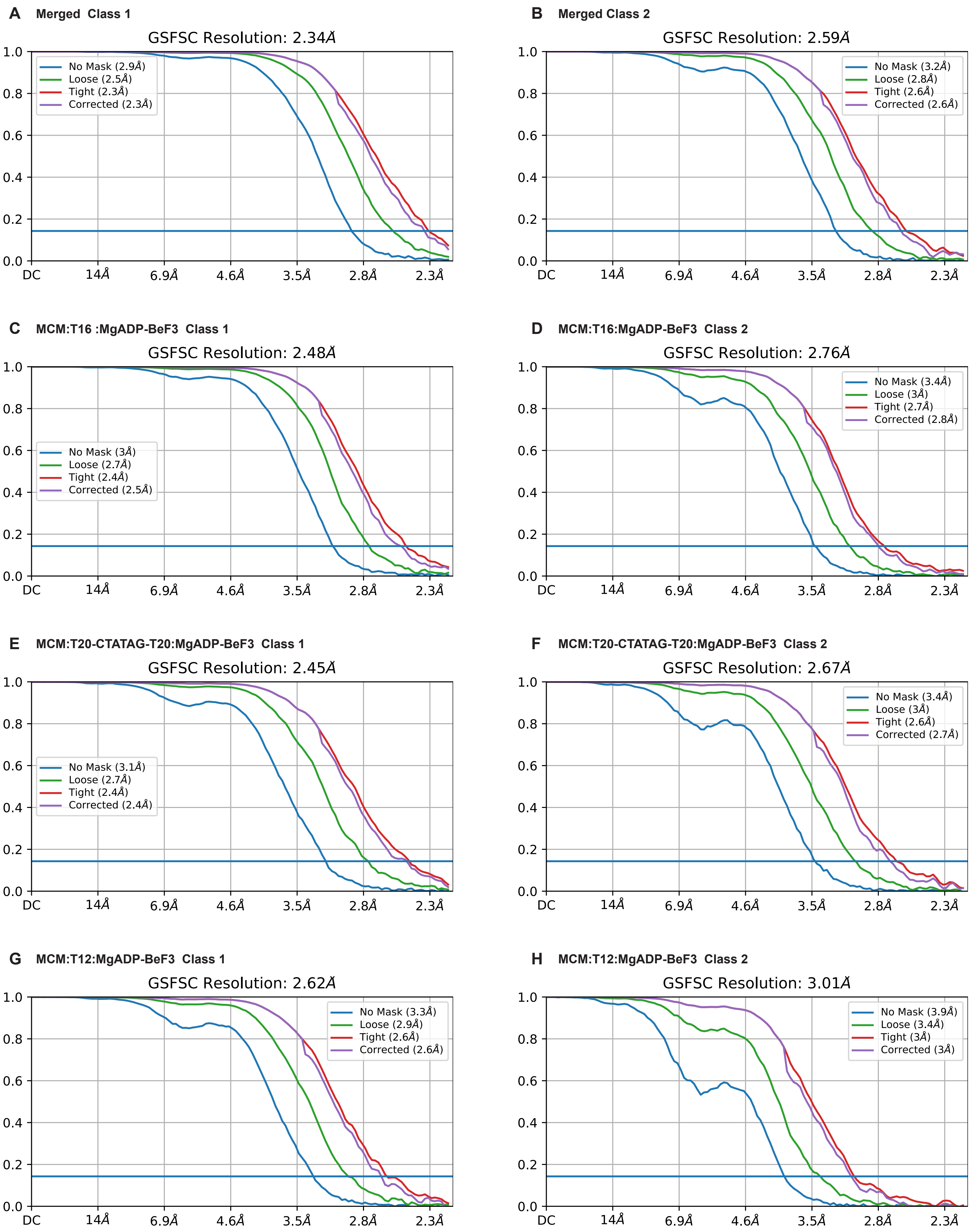

Figure S1. Gold-Standard Fourier Shell Correlation for the final refinements of each structure reported. **A.** Combined class 1 particles for all three distinct samples (MCM:T16:MgADP-BeF_3, MCM:T20-CTATAG-T20:MgADP-BeF_3, MCM:T12:MgADP-BeF_3). **B.** Combined class 2 particles for all three distinct samples (MCM:T16:MgADP-BeF_3, MCM:T20-CTATAG-T20:MgADP-BeF_3, MCM:T12:MgADP-BeF_3). **C.** MCM:T16:MgADP-BeF_3 class 1. **D.** MCM:T16:MgADP-BeF_3 class 2. **E.** MCM:T20-CTATAG-T20:MgADP-BeF_3 class 1. **F.** MCM:T20-CTATAG-T20:MgADP-BeF_3 class 2. **G.** MCM:T12:MgADP-BeF_3 class 1. **H.** MCM:T12:MgADP-BeF_3 class 2.

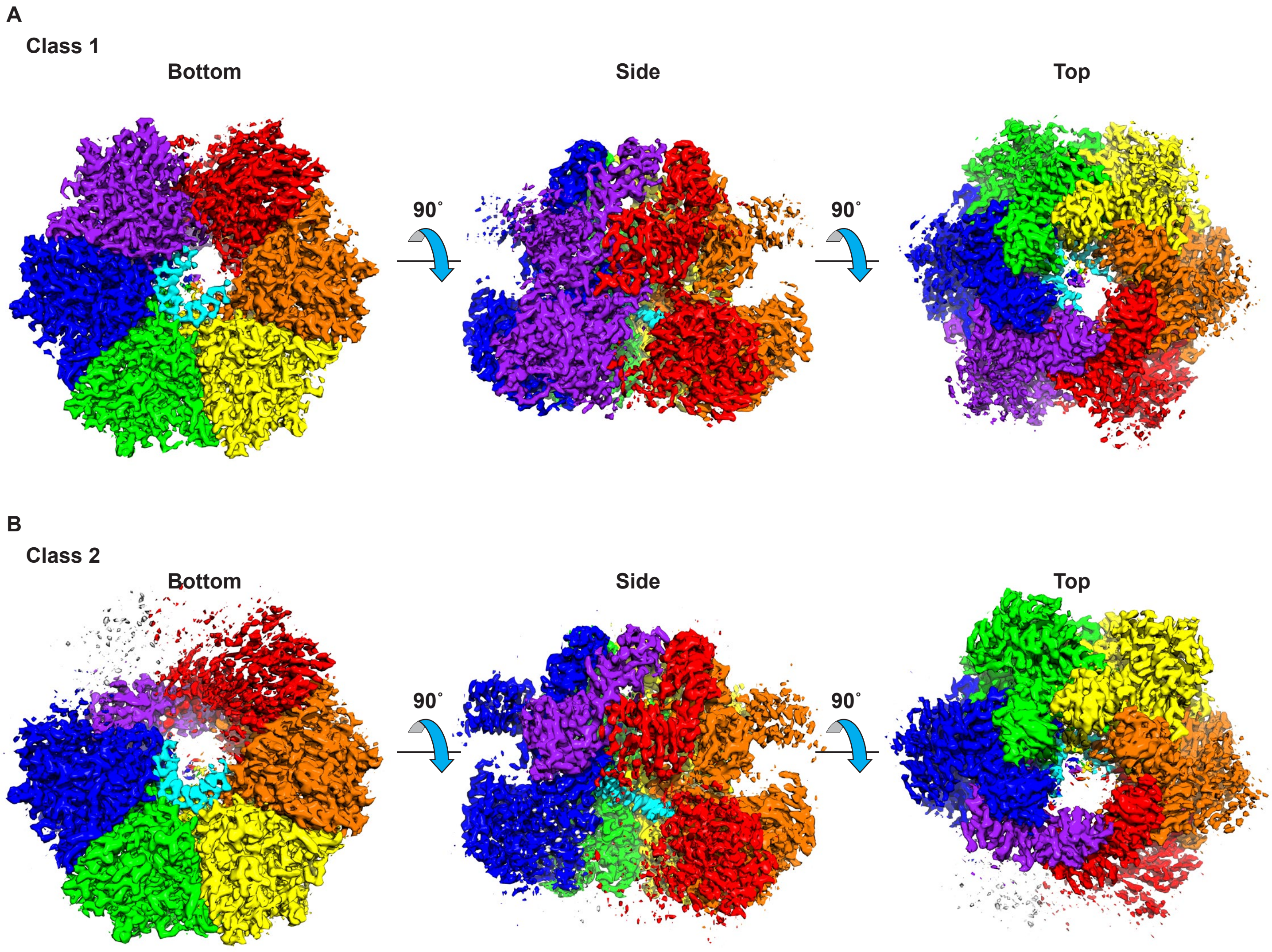

Figure S2. Overall architectures of the two structural classes of MCM:T16:MgADP-BeF_3. Three orthogonal, consistent views are illustrated for the two classes. The illustrated maps were obtained by refinement of the respective classes obtained with a 16-mer poly-dT oligonucleotide (see Table 1). Both Classes consist of a two-tiered hexameric ring that binds single-stranded DNA at the larger ATPase tier. The defined views place the N-terminal tier at the top of the complex and the ATPase tier at the bottom. Each figure panel was prepared with Chimera[1]. **A.** The final sharpened map following homogeneous refinement of class 1 is colored by subunit proximity to the atomic model coordinate file. Class 1 has 6 well-defined domains at both tiers. The peripheral helical bundle subdomains of the N-terminal tier appear less ordered than the other subdomains. **B.** The final sharpened map following homogeneous refinement of class 2 is colored by subunit proximity to the atomic model coordinate file. Class 2 has 6 well-defined domains at the center of the N-terminal tier and 5 well-defined ATPase domains. The ATPase domain of the purple subunit is poorly ordered. Two of the peripheral helical bundle domains of the N-terminal tier are poorly ordered. Maps are displayed by Chimera [1] at contour level 1.4 for class 1 and at contour level 0.9 for class 2 to emphasize the well-ordered DNA-bound portion.

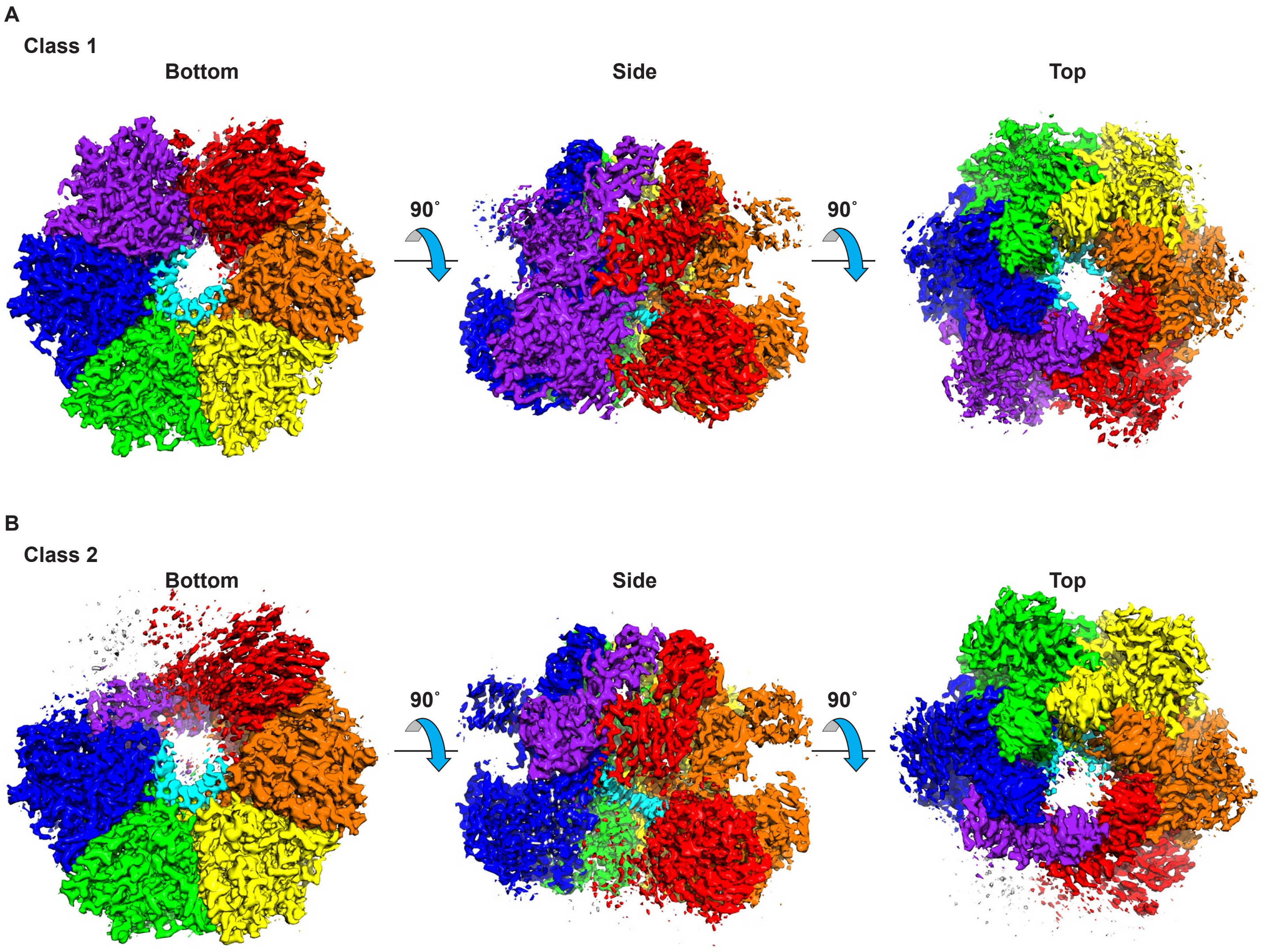

Figure S3. Overall architectures of the two structural classes of MCM:T20-CTATAG-T20:MgADP-BeF_3. Three orthogonal, consistent views are illustrated for the two classes. The illustrated maps were obtained by refinement of the particles of the respective classes obtained with an "X-shaped" oligonucleotide. Both Classes consist of a two-tiered hexameric ring that binds single-stranded DNA at the larger ATPase tier. The defined views place the N-terminal tier at the top of the complex and the ATPase tier at the bottom. Each figure panel was prepared with Chimera[1]. **A.** The final sharpened map following homogeneous refinement of class 1 is colored by subunit proximity to the atomic model coordinate file. Class 1 has 6 well-defined domains at both tiers. The peripheral helical bundle subdomains of the N-terminal tier appear less ordered than the other subdomains. **B**. The final sharpened map following homogeneous refinement of class 2 is colored by subunit proximity to the atomic model coordinate file. Class 2 has 6 well-defined domains at the center of the N-terminal tier and 5 well-defined ATPase domains. The ATPase domain of the purple subunit is poorly ordered. Two of the peripheral helical bundle domains of the N-terminal tier are poorly ordered. Maps are displayed by Chimera [1] at contour level 1.4 for class 1 and at contour level 1.1 for class 2 to emphasize the well-ordered DNA-bound portion.

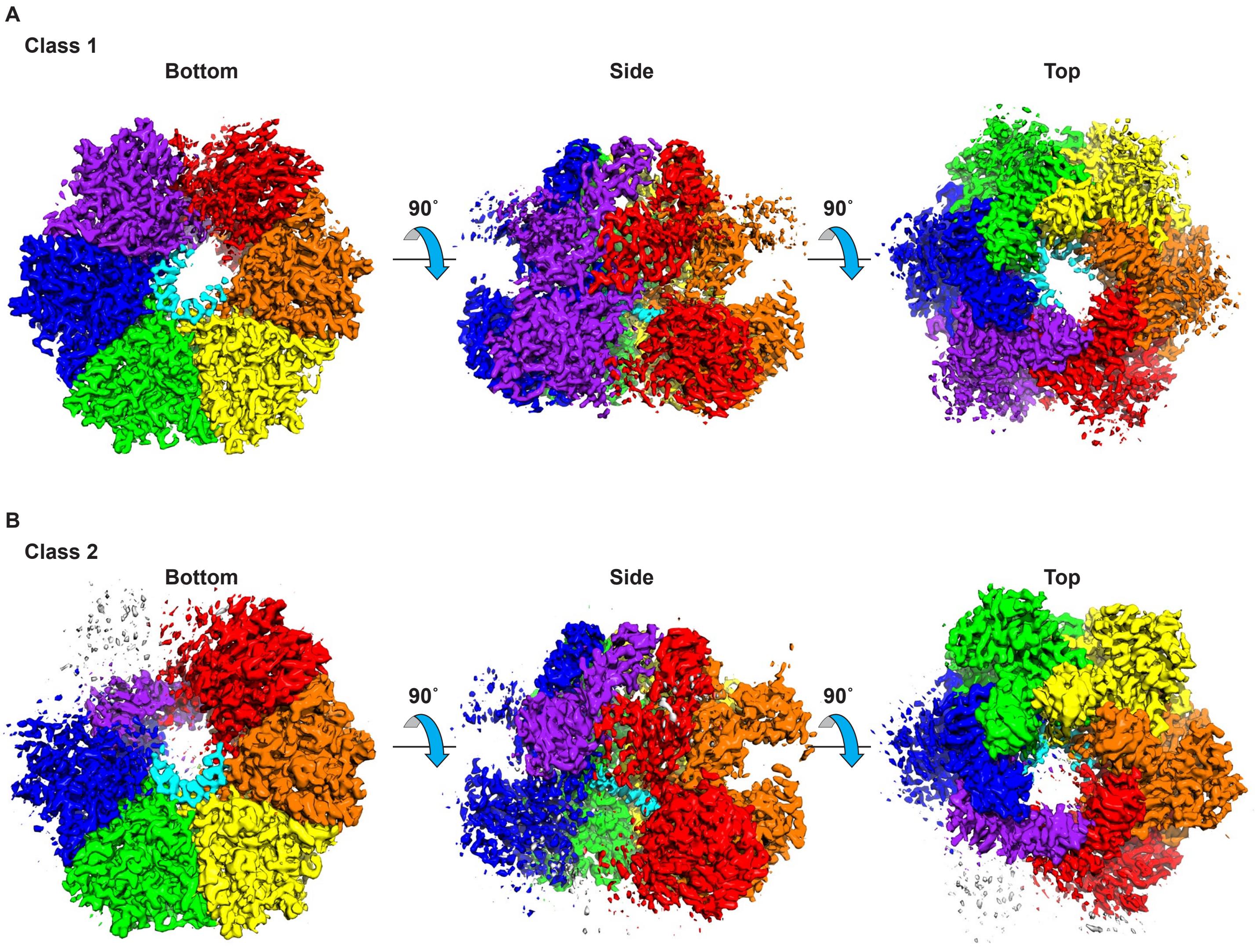

Figure S4. Overall architectures of the two structural classes of MCM:T12:MgADP-BeF_3. Three orthogonal, consistent views are illustrated for the two classes. The illustrated maps were obtained by refinement of the respective classes obtained with a 12-mer poly-dT oligonucleotide. Both Classes consist of a two-tiered hexameric ring that binds single-stranded DNA at the larger ATPase tier. The defined views place the N-terminal tier at the top of the complex and the ATPase tier at the bottom. Each figure panel was prepared with Chimera[1]. **A.** The final sharpened map following homogeneous refinement of class 1 is colored by subunit proximity to the atomic model coordinate file. Class 1 has 6 well-defined domains at both tiers. The peripheral helical bundle subdomains of the N-terminal tier appear less ordered than the other subdomains. **B.** The final sharpened map following homogeneous refinement of class 2 is colored by subunit proximity to the atomic model coordinate file. Class 2 has 6 well-defined domains at the center of the N-terminal tier and 5 well-defined ATPase domains. The ATPase domain of the purple subunit is poorly ordered. Two of the peripheral helical bundle domains of the N-terminal tier are poorly ordered. Maps are displayed by Chimera [1] at contour level 1.4 for class 1 and at contour level 0.9 for class 2 to emphasize the well-ordered DNA-bound portion.

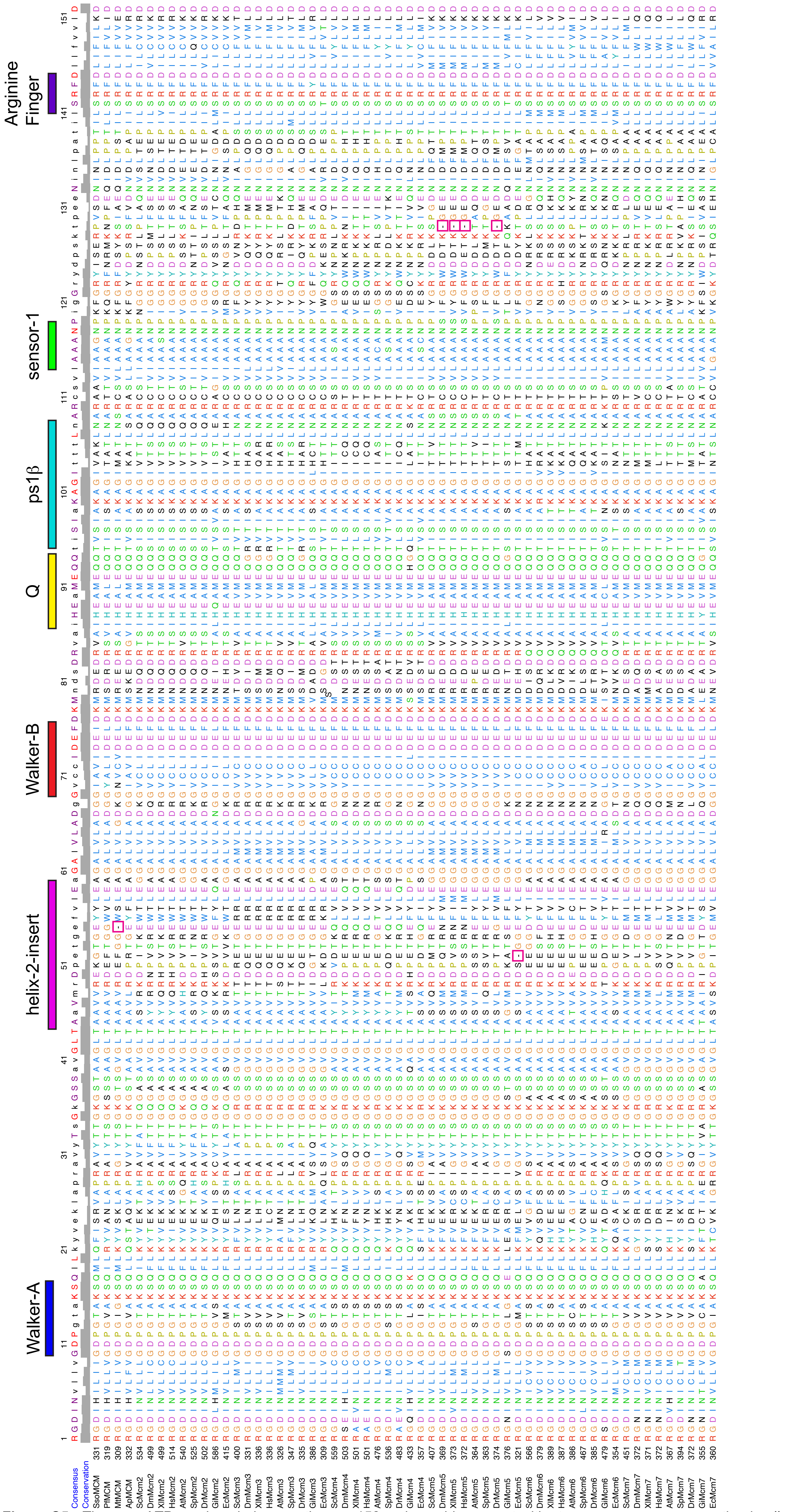

Figure S5. The core ATPase domain is a contiguous 152-amino acid segment with strong sequence conservation in all archaeal and eukaryotic MCM proteins. Of 58 sequences, 52 possess this 152-amino acid configuration. Six have a one-residue deletion (magenta boxes) for a 151-amino acid core ATPase. See Figure 2. Sso=*Saccharolobus solfataricus*; Pf=*Pyrococcus furiosus*; Mt=*Methanothermobacter thermautotrophicus*; Ap=*Aeropyrum pernix*; Sc=*Saccharomyces cerevisiae*; Dm=*Drosophila melanogaster*; Xl=*Xenopus laevis*; Hs=*Homo sapiens*; At=*Arabidopsis thaliana*; Sp=*Schizosaccharomyces pombe*; Dr=*Danio rerio*; Gi=*Giardia lamblia*; Ec=*Encephalitozoon cuniculi*.

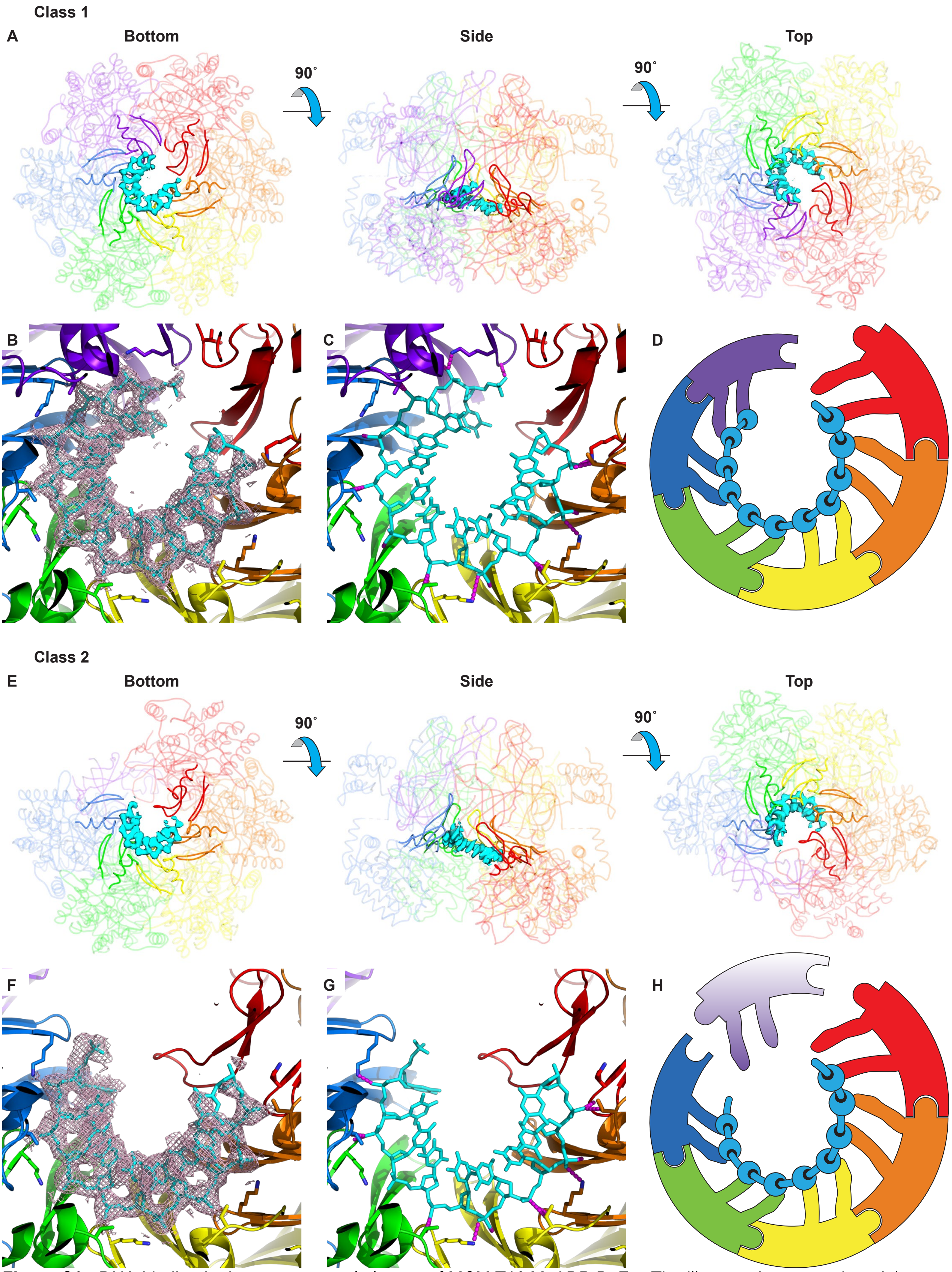

Figure S6. DNA-binding in the two structural classes of MCM:T16:MgADP-BeF_3. The illustrated maps and models were obtained by refinement of the respective classes obtained with a 16-mer poly-dT oligonucleotide. **A.** Three orthogonal views of class 1 with the ssDNA portion of the map depicted in cyan and the protein model in ribbon representation. The two DNA-binding hairpins of each subunit are shown in opaque, and the other protein features are partially transparent. The panel view is identical to Fig. 1A-B. **B.** Zoom-in view of the class 1 model illustrating the DNA-binding hairpins and ssDNA. All 4.0-sigma density within 2.5 Å of the modeled DNA shown in mesh. Eleven nucleotides of ssDNA are observed for class 1. **C.** An identical view to panel B illustrating all MCM:DNA hydrogen bonding interactions (< 3.5 Å) in dashed magenta with density mesh removed for clarity. **D.** Cartoon summarizing the sequential interactions of MCM DNA-binding hairpins with the 11 DNA nucleotides, each represented by a bead on a string. **E.** Three orthogonal views of class 2 with the ssDNA portion of the map depicted in cyan and the protein model in ribbon representation. The two DNA-binding hairpins of each subunit are shown in opaque, and the other protein features are partially transparent. The panel view is identical to Fig. 1A-B. **F.** Zoom-in view of the class 2 model illustrating the DNA-binding hairpins and ssDNA. All 4.0-sigma density within 2.5 Å of the modeled DNA shown in mesh. Nine nucleotides of ssDNA are observed for class 2. **G.** An identical view to panel B illustrating all MCM:DNA hydrogen bonding interactions (< 3.5 Å) in dashed magenta with density mesh removed for clarity. **H.** Cartoon summarizing the sequential interactions of MCM DNA-binding hairpins with the 9 DNA nucleotides, each represented by a bead on a string. The poorly ordered purple subunit is represented with gradient shading. Panels A and E were prepared with Chimera [1], and panels C, D, F and G were prepared with Pymol [2].

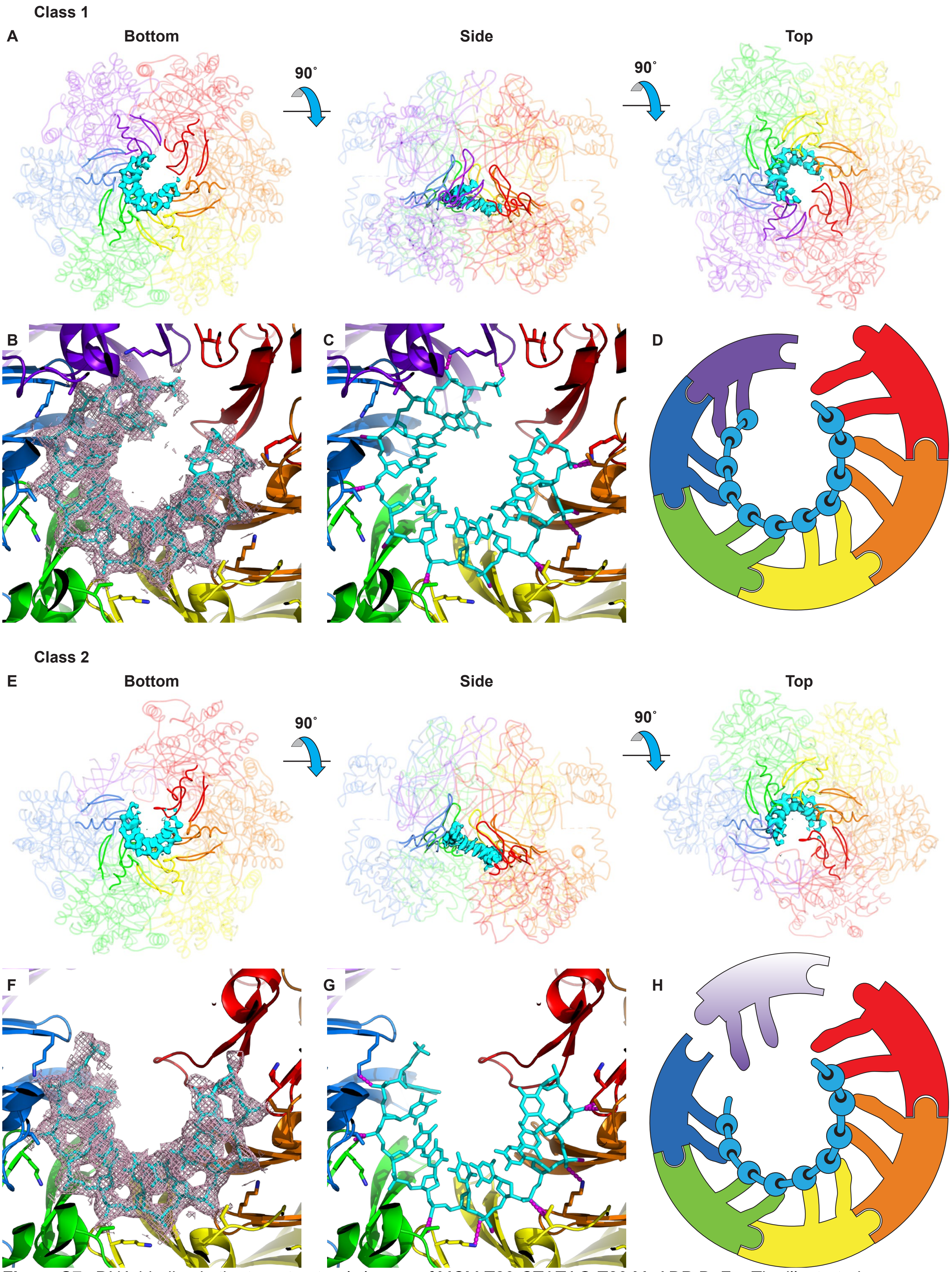

Figure S7. DNA-binding in the two structural classes of MCM:T20-CTATAG-T20:MgADP-BeF_3. The illustrated maps were obtained by refinement of the particles of the respective classes obtained with an "X-shaped" oligonucleotide (see Table 1). **A.** Three orthogonal views of class 1 with the ssDNA portion of the map depicted in cyan and the protein model in ribbon representation. The two DNA-binding hairpins of each subunit are shown in opaque, and the other protein features are partially transparent. The panel view is identical to Fig. 1A-B. **B.** Zoom-in view of the class 1 model illustrating the DNA-binding hairpins and ssDNA. All 4.0-sigma density within 2.5 Å of the modeled DNA shown in mesh. Eleven nucleotides of ssDNA are observed for class 1. **C.** An identical view to panel B illustrating all MCM:DNA hydrogen bonding interactions (< 3.5 Å) in dashed magenta with density mesh removed for clarity. **D.** Cartoon summarizing the sequential interactions of MCM DNA-binding hairpins with the 11 DNA nucleotides, each represented by a bead on a string. **E.** Three orthogonal views of class 2 with the ssDNA portion of the map depicted in cyan and the protein model in ribbon representation. The two DNA-binding hairpins of each subunit are shown in opaque, and the other protein features are partially transparent. The panel view is identical to Fig. 1A-B. **F.** Zoom-in view of the class 2 model illustrating the DNA-binding hairpins and ssDNA. All 4.0-sigma density within 2.5 Å of the modeled DNA shown in mesh. Nine nucleotides of ssDNA are observed for class 2. **G.** An identical view to panel B illustrating all MCM:DNA hydrogen bonding interactions (< 3.5 Å) in dashed magenta with density mesh removed for clarity. **H.** Cartoon summarizing the sequential interactions of MCM DNA-binding hairpins with the 9 DNA nucleotides, each represented by a bead on a string. The poorly ordered purple subunit is represented with gradient shading. Panels A and E were prepared with Chimera [1], and panels C, D, F and G were prepared with Pymol [2].

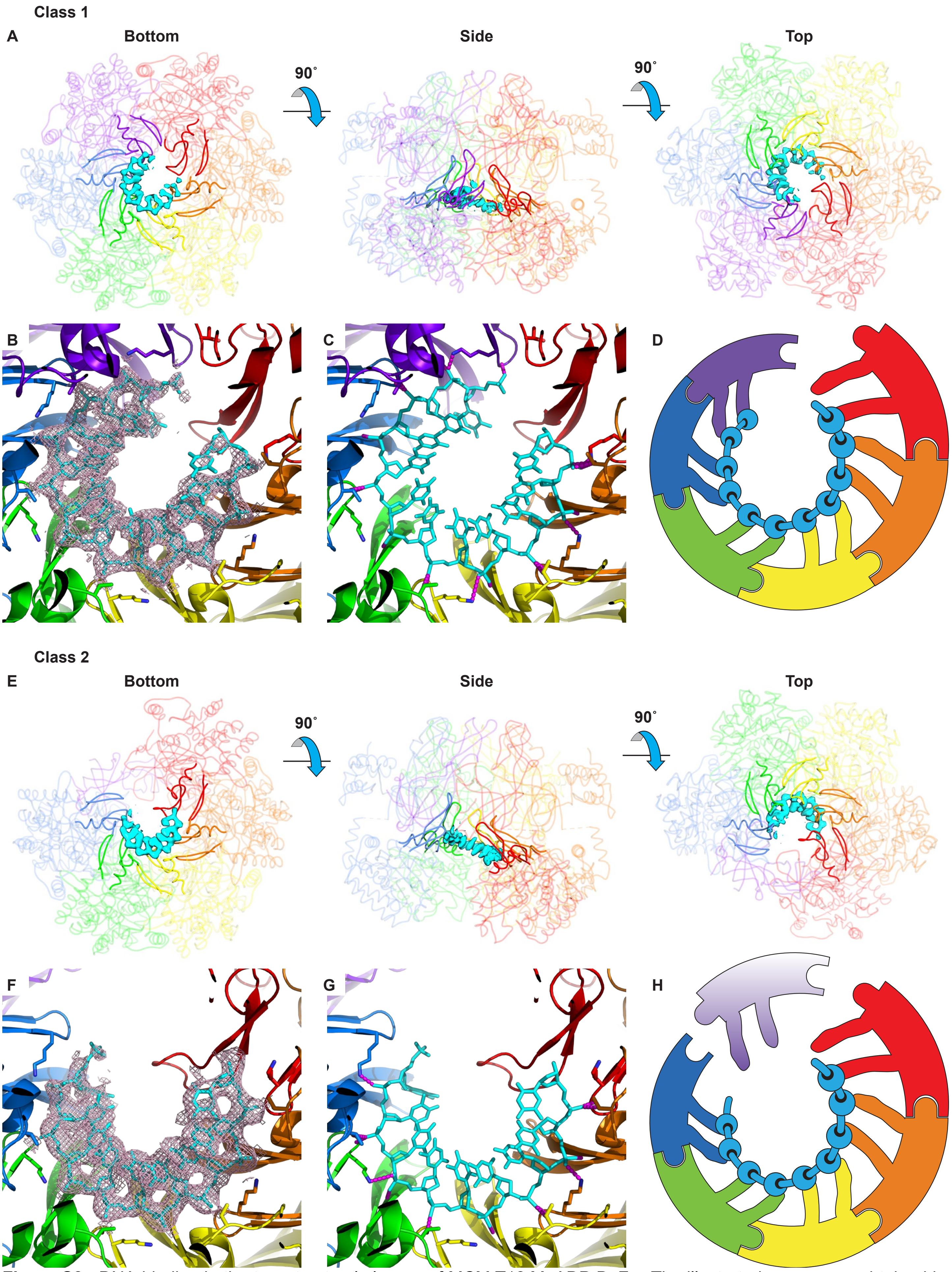

Figure S8. DNA-binding in the two structural classes of MCM:T12:MgADP-BeF₃. The illustrated maps were obtained by refinement of the respective classes obtained with a 12-mer poly-dT oligonucleotide (see Table 1). **A.** Three orthogonal views of class 1 with the ssDNA portion of the map depicted in cyan and the protein model in ribbon representation. The two DNA-binding hairpins of each subunit are shown in opaque, and the other protein features are partially transparent. The panel view is identical to Fig. 1A-B. **B.** Zoom-in view of the class 1 model illustrating the DNA-binding hairpins and ssDNA. All 4.0-sigma density within 2.5 Å of the modeled DNA shown in mesh. Eleven nucleotides of ssDNA are observed for class 1. **C.** An identical view to panel B illustrating all MCM:DNA hydrogen bonding interactions (< 3.5 Å) in dashed magenta with density mesh removed for clarity. **D.** Cartoon summarizing the sequential interactions of MCM DNA-binding hairpins with the 11 DNA nucleotides, each represented by a bead on a string. **E.** Three orthogonal views of class 2 with the ssDNA portion of the map depicted in cyan and the protein model in ribbon representation. The two DNA-binding hairpins of each subunit are shown in opaque, and the other protein features are partially transparent. The panel view is identical to Fig. 1A-B. **F.** Zoom-in view of the class 2 model illustrating the DNA-binding hairpins and ssDNA. All 4.0-sigma density within 2.5 Å of the modeled DNA shown in mesh. Nine nucleotides of ssDNA are observed for class 2. **G.** An identical view to panel B illustrating all MCM:DNA hydrogen bonding interactions (< 3.5 Å) in dashed magenta with density mesh removed for clarity. **H.** Cartoon summarizing the sequential interactions of MCM DNA-binding hairpins with the 9 DNA nucleotides, each represented by a bead on a string. The poorly ordered purple subunit is represented with gradient shading. Panels A and E were prepared with Chimera [1], and panels C, D, F and G were prepared with Pymol [2].

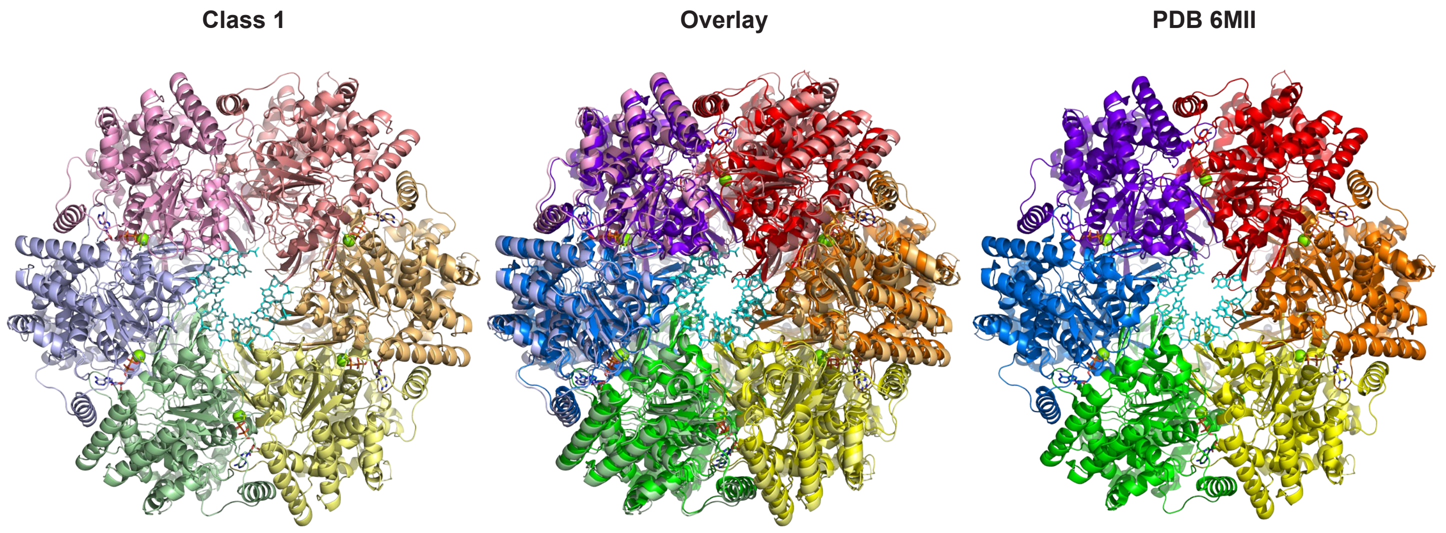

Figure S9. Comparison of the structures of the Cryo-EM Class 1 structure with the crystal structure of the complex (PDB 6MII [3]. Both structures bind encircled ssDNA in an equivalent spiral staircase mode. The central channel of the crystal structure is slightly more constricted, principally because the ATPase domains colored orange and red are slightly closer to the channel center. Figure panels were prepared with Pymol [2].

A

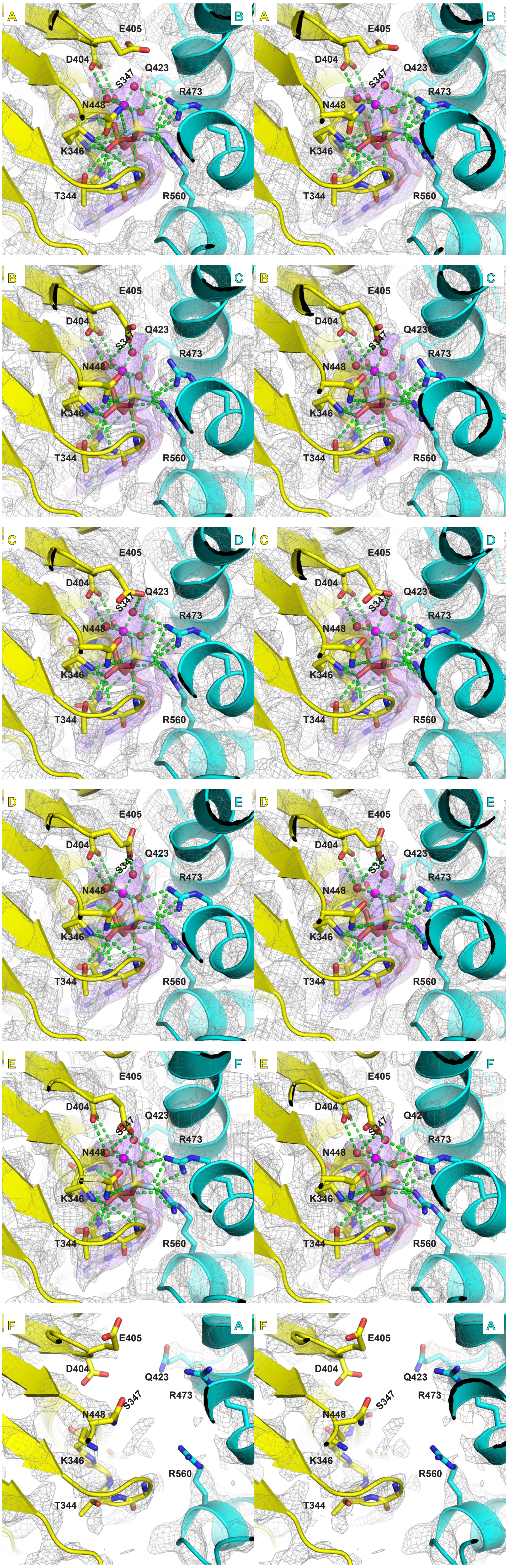

B

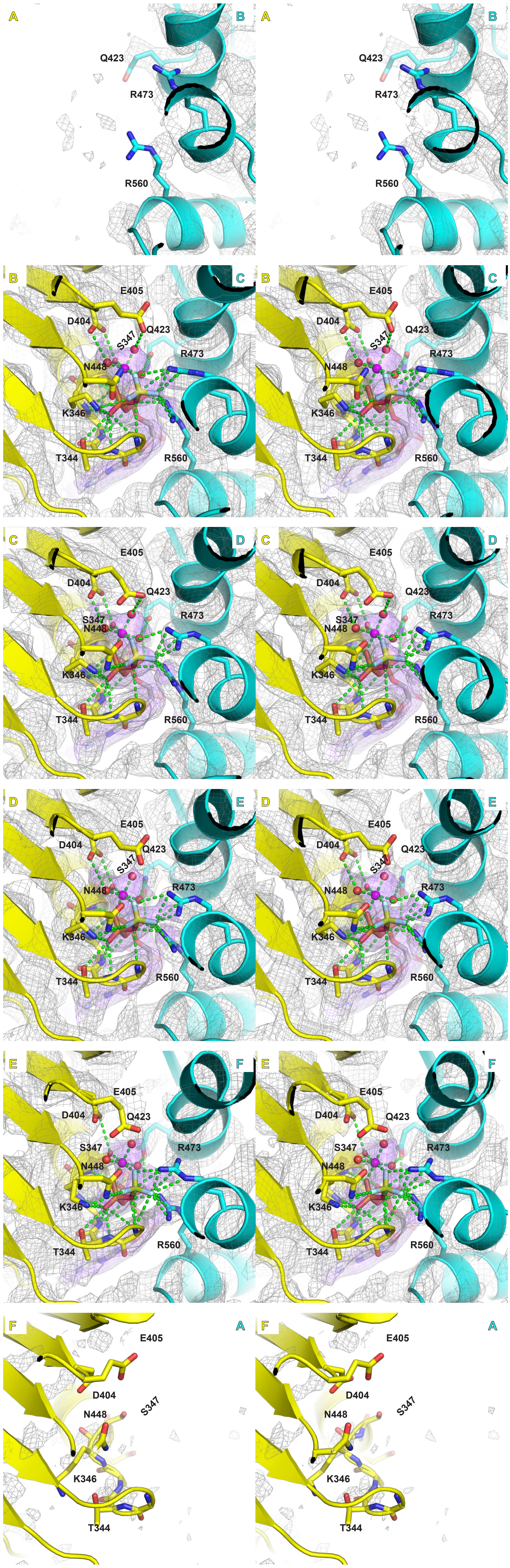

A
B
Q423
R473
R560
B
C
E405
D404
S347
Q423
R473
N448
K346
T344
R560
C
D
E405
D404
Q423
S347
R473
N448
K346
T344
R560
D
E
E405
D404
Q423
S347
N448
R473
K346
T344
R560
E
F
E405
D404
Q423
S347
R473
N448
K346
T344
R560
F
A
E405
D404
N448
S347
K346
T344

Figure S10. Stereoviews of the subunit interfaces of the two structural classes of MCM:T16:MgADP-BeF_3. The illustrated maps and models were obtained by refinement of the respective classes obtained with a 16-mer poly-dT oligonucleotide (see Table 1). For each view, the subunit containing the Walker-A and -B residues is depicted in yellow, and the neighboring subunit with the arginine finger is depicted in cyan. Interactions involving Mg/ATP are depicted with green dashes. Interacting side-chains are shown in stick and labeled: T344, K346, and S347 of Walker-A; D404 and E405 of Walker-B; N448 of Sensor-1; R560 of Sensor-2; R473 arginine finger; and Q423. The magnesium ion in magenta has an octahedral coordination sphere derived from an oxygen of the β-phosphate, a fluoride of the BeF_3 moiety, the conserved oxygen side-chain of S347, and three water molecules. Although functionally critical, the side-chain for Walker-B E405 is not well-ordered. **A.** The illustrated maps and models were obtained by refinement of the particles of class 1. Density above 5.0-sigma is shown in grey mesh with density within 2.5 Å of the modeled Mg/ATP also illustrated in purple transparent surface. Five interfaces show strong nucleotide density that do not strongly differ from each other. The sixth interface (F/A) is more open and does not show significant nucleotide density. **B.** The illustrated maps and models were obtained by refinement of the particles of class 2. Density above 5.0-sigma is shown in grey mesh with density within 2.5 Å of the modeled Mg/ATP also illustrated in purple transparent surface. Four interfaces show strong nucleotide density that do not strongly differ from each other. The ATPase domain of chain A is poorly ordered for class 2, and hence two of the interfaces (A/B and F/A) do not have a well-ordered Mg/ATP site.

A

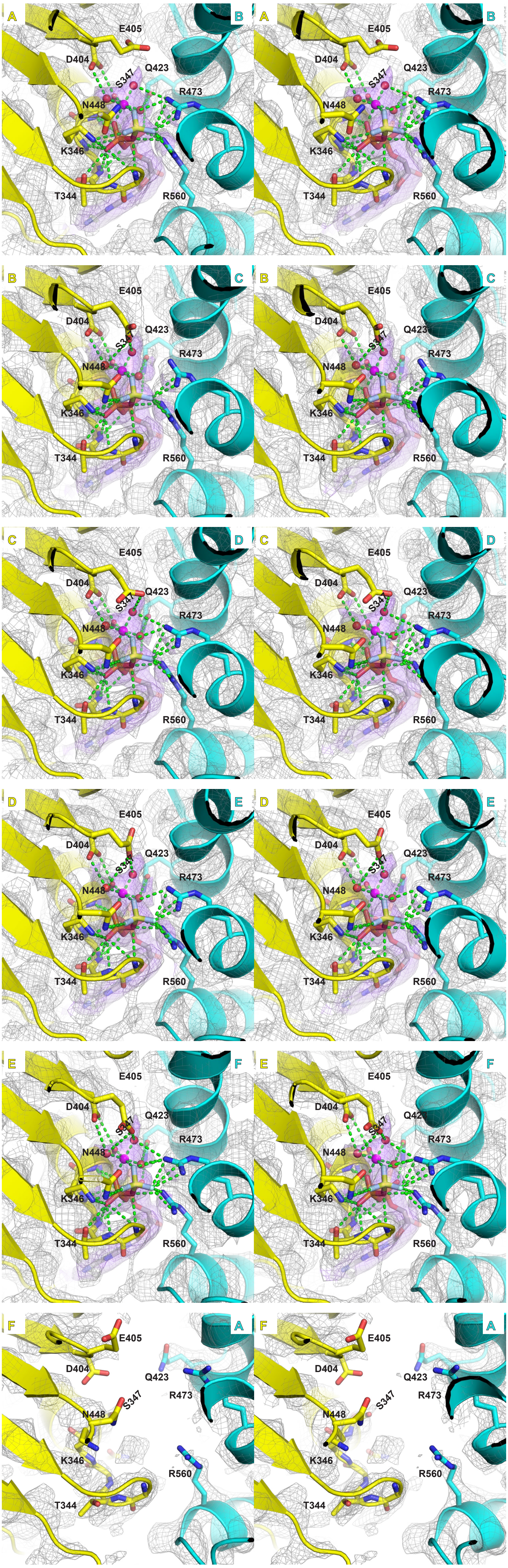

B

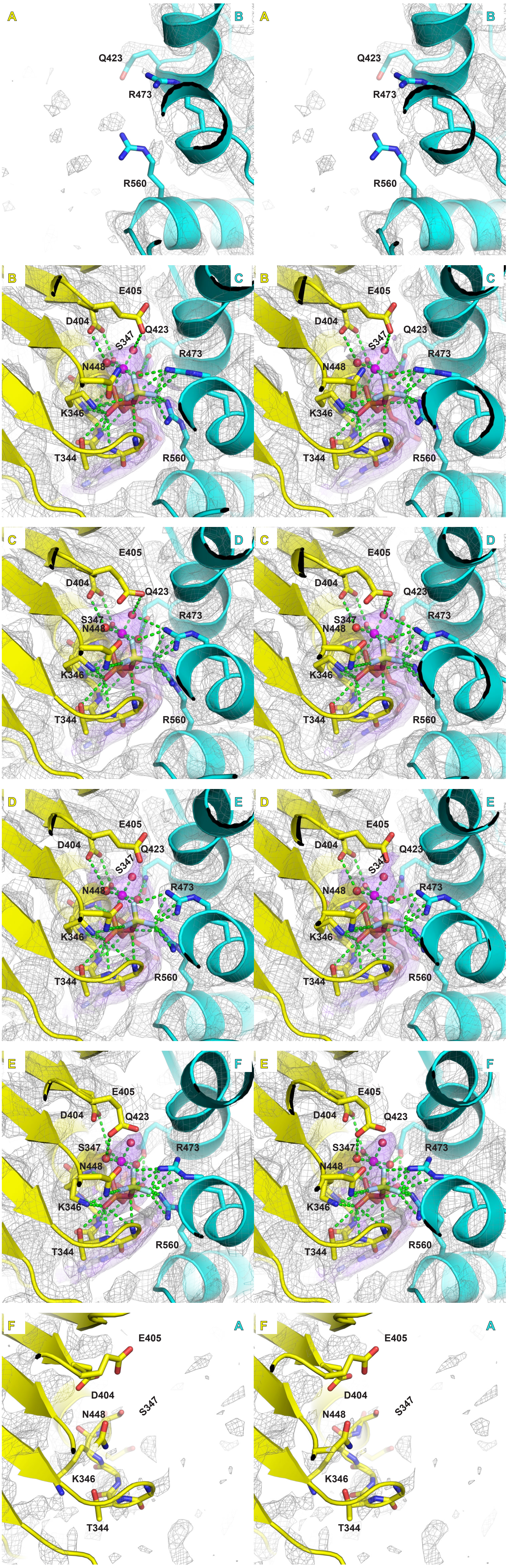

A
B
Q423
R473
R560
B
C
E405
D404
S347
Q423
N448
R473
K346
T344
R560
C
D
E405
D404
Q423
S347
N448
R473
K346
T344
R560
D
E
E405
D404
Q423
S347
N448
R473
K346
T344
R560
E
F
E405
D404
Q423
S347
N448
R473
K346
T344
R560
F
A
E405
D404
N448
S347
K346
T344

Figure S11. Stereoviews of the subunit interfaces of the two structural classes of MCM:T20-CTATAG-T20:MgADP-BeF_3. The illustrated maps and models were obtained by refinement of the particles of the respective classes obtained with an "X-shaped" oligonucleotide (see Table 1). For each view, the subunit containing the Walker-A and -B residues is depicted in yellow, and the neighboring subunit with the arginine finger is depicted in cyan. Interactions involving Mg/ATP are depicted with green dashes. Interacting side-chains are shown in stick and labeled: T344, K346, and S347 of Walker-A; D404 and E405 of Walker-B; N448 of Sensor-1; R560 of Sensor-2; R473 arginine finger; and Q423. The magnesium ion in magenta has an octahedral coordination sphere derived from an oxygen of the β-phosphate, a fluoride of the BeF_3 moiety, the conserved oxygen side-chain of S347, and three water molecules. Although functionally critical, the side-chain for Walker-B E405 is not well-ordered. **A.** The illustrated maps and models were obtained by refinement of the particles of class 1. Density above 5.0-sigma is shown in grey mesh with density within 2.5 Å of the modeled Mg/ATP also illustrated in purple transparent surface. Five interfaces show strong nucleotide density that do not strongly differ from each other. The sixth interface (F/A) is more open and does not show significant nucleotide density. **B.** The illustrated maps and models were obtained by refinement of the particles of class 2. Density above 5.0-sigma is shown in grey mesh with density within 2.5 Å of the modeled Mg/ATP also illustrated in purple transparent surface. Four interfaces show strong nucleotide density that do not strongly differ from each other. The ATPase domain of chain A is poorly ordered for class 2, and hence two of the interfaces (A/B and F/A) do not have a well-ordered Mg/ATP site.

A

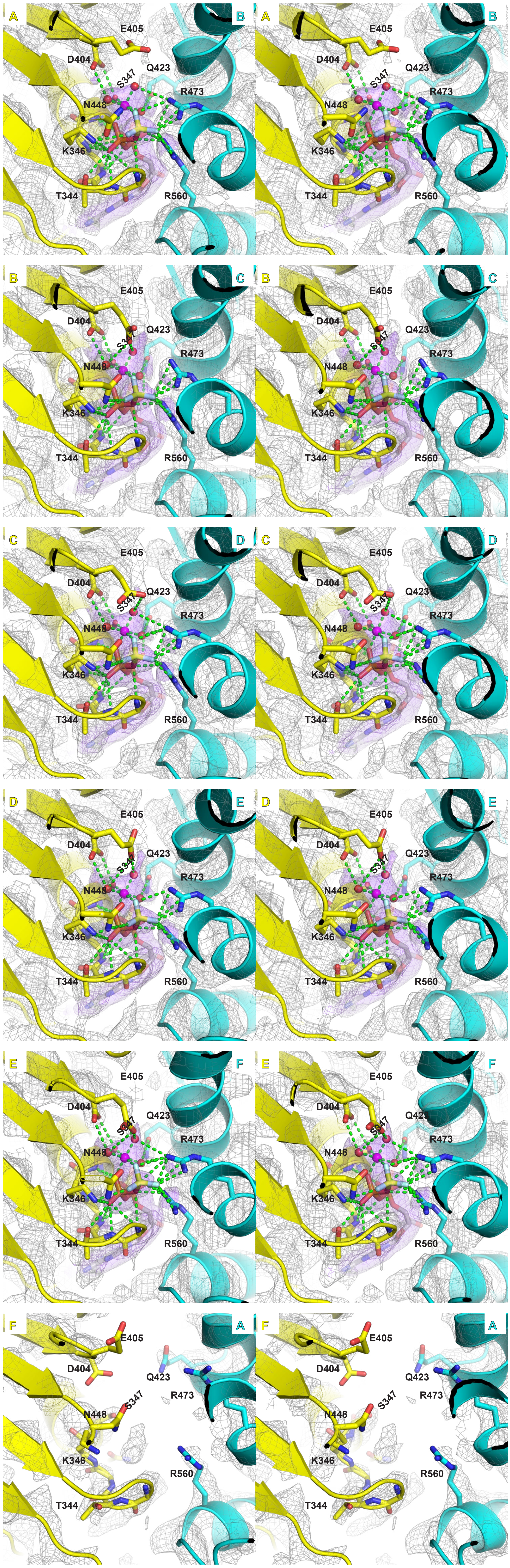

B

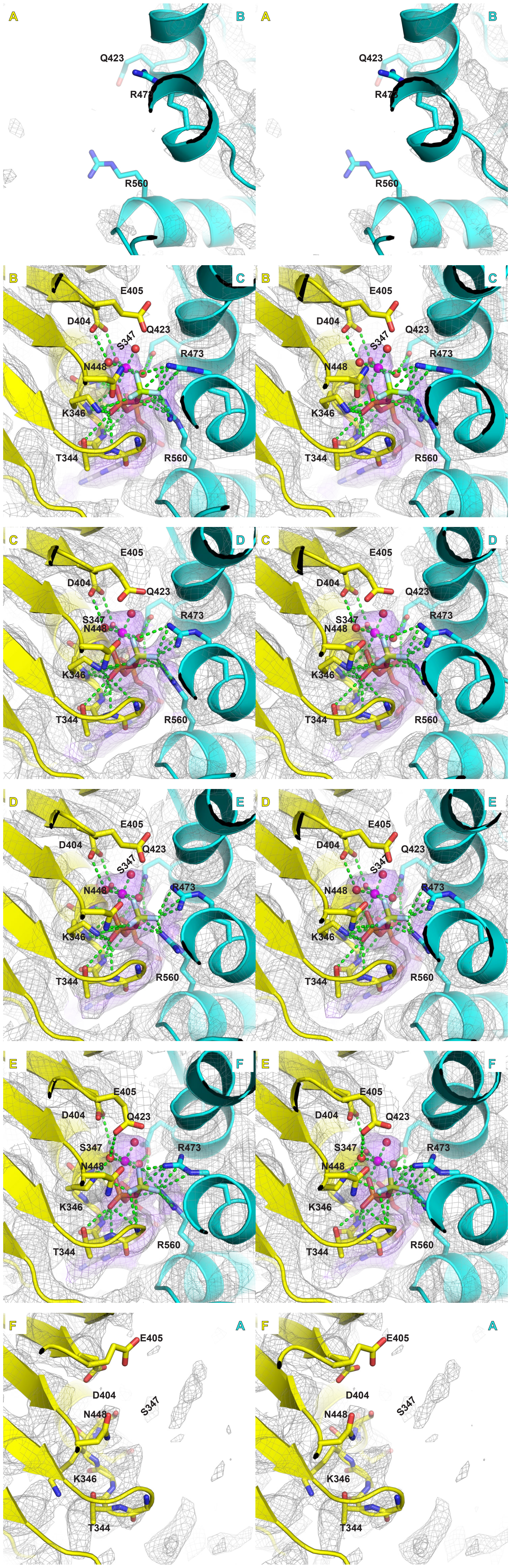

Fig. S12. Stereoviews of the subunit interfaces of the two structural classes of MCM:T12:MgADP-BeF_3. The illustrated maps and models were obtained by refinement of the respective classes obtained with a 12-mer poly-dT oligonucleotide (see Table 1). For each view, the subunit containing the Walker-A and -B residues is depicted in yellow, and the neighboring subunit with the arginine finger is depicted in cyan. Interactions involving Mg/ATP are depicted with green dashes. Interacting side-chains are shown in stick and labeled: T344, K346, and S347 of Walker-A; D404 and E405 of Walker-B; N448 of Sensor-1; R560 of Sensor-2; R473 arginine finger; and Q423. The magnesium ion in magenta has an octahedral coordination sphere derived from an oxygen of the β-phosphate, a fluoride of the BeF_3 moiety, the conserved oxygen side-chain of S347, and three water molecules. Although functionally critical, the side-chain for Walker-B E405 is not well-ordered. **A.** The illustrated maps and models were obtained by refinement of the particles of class 1. Density above 5.0-sigma is shown in grey mesh with density within 2.5 Å of the modeled Mg/ATP also illustrated in purple transparent surface. Five interfaces show strong nucleotide density that do not strongly differ from each other. The sixth interface (F/A) is more open and does not show significant nucleotide density. **B.** The illustrated maps and models were obtained by refinement of the particles of class 2. Density above 5.0-sigma is shown in grey mesh with density within 2.5 Å of the modeled Mg/ATP also illustrated in purple transparent surface. Four interfaces show strong nucleotide density that do not strongly differ from each other. The ATPase domain of chain A is poorly ordered for class 2, and hence two of the interfaces (A/B and F/A) do not have a well-ordered Mg/ATP site.

Movie Captions

Movie 1. Translocation mechanism. Left: Oscillation between DNA-engagement/DNA-disengagement illustrated by the maps of the two classes of structure reported here. The structure is colored by subunit proximity and viewed from the bottom of the complex (ATPase tier side). These oscillations were sequentially permuted around the ring to illustrate a sequential rotary mechanism. Images were prepared with Chimera [1] and converted to a movie with Adobe Premiere Pro. Right: Cartoon representation of the translocation depicting the two hairpins of each subunit and individual nucleotides of DNA as beads on a string. The poorly ordered ATPase domain is in gradient shade. Please view as a loop.

Movie 2. Three orthogonal views of the translocation mechanism. The EM density for the two classes was morphed in Chimera [1] to illustrate the transitions between DNA-engagement and DNA-disengagement revealed by the cryo-EM density. The transitions were sequentially permuted around the ring to illustrate a sequential rotary mechanism as in Movie 1. Images were rendered with Chimera [1] and converted to a movie with Adobe Premiere Pro. Please view as a loop.

Movie 3. The core MCM ATPase domains of eukaryotes are archaea are highly similar in structure. **Left**, the 152-amino acid core ATPase domain structures are superimposed and colored by subunit. HsMcm2-7 subunit structures are from PDB 6XTX [4]. The core structure is highly conserved in structure with the most prominent variability in the helix-2-insert hairpins of *Hs*Mcm2 and *Hs*Mcm5, which occupy the “top” and “bottom” positions of the staircase (see Figure 6A) and are thus anticipated to be more mobile. **Right,** the superimposed structures are colored according to their DNA-binding and ATPase motifs (see Figure 6 and Figure S5). Images were rendered with Pymol [2] and converted to a movie with Adobe Premiere Pro. Please view as a loop.

References

1. Pettersen, E. F.; Goddard, T. D.; Huang, C. C.; Couch, G. S.; Greenblatt, D. M.; Meng, E. C.; Ferrin, T. E., UCSF Chimera--a visualization system for exploratory research and analysis. *J Comput Chem* **2004,** 25, (13), 1605-12.
2. Schrodinger, LLC *The PyMOL Molecular Graphics System, Version 1.3r1*, 2010.
3. Meagher, M.; Epling, L. B.; Enemark, E. J., DNA translocation mechanism of the MCM complex and implications for replication initiation. *Nat Commun* **2019,** 10, (1), 3117.
4. Rzechorzek, N. J.; Hardwick, S. W.; Jatikusumo, V. A.; Chirgadze, D. Y.; Pellegrini, L., CryoEM structures of human CMG-ATPgammaS-DNA and CMG-AND-1 complexes. *Nucleic Acids Res* **2020,** 48, (12), 6980-6995.
